# Chemical Evaluation, Antioxidant, Antiproliferative, Anti-Inflammatory and Antibacterial Activities of Organic Extract and Semi-Purified Fractions of the Adriatic Sea Fan, *Eunicella cavolini*

**DOI:** 10.3390/molecules26195751

**Published:** 2021-09-23

**Authors:** Dario Matulja, Petra Grbčić, Krunoslav Bojanić, Natalija Topić-Popović, Rozelindra Čož-Rakovac, Sylvain Laclef, Tomislav Šmuc, Ozren Jović, Dean Marković, Sandra Kraljević Pavelić

**Affiliations:** 1 Department of Biotechnology, University of Rijeka, Radmile Matejčić 2, 51000 Rijeka, Croatia; dario.matulja@biotech.uniri.hr (D.M.); petra.grbcic@biotech.uniri.hr (P.G.); 2 Ruđer Bošković Institute, Bijenička Cesta 54, 10000 Zagreb, Croatia; Krunoslav.Bojanic@irb.hr (K.B.); Natalija.Topic.Popovic@irb.hr (N.T.-P.); Rozelindra.Coz-Rakovac@irb.hr (R.Č.-R.); tomislav.smuc@irb.hr (T.Š.); ozren.jovic@irb.hr (O.J.); 3 Laboratoire de Glycochimie, des Antimicrobiens et des Agroressources (LG2A) UMR CNRS 7378—Institut de Chimie de Picardie FR 3085, Université de Picardie Jules Verne, 33 Rue Saint Leu, 80039 Amiens, France; sylvain.laclef@u-picardie.fr; 4 Faculty of Health Studies, University of Rijeka, Viktora Cara Emina 5, 51000 Rijeka, Croatia

**Keywords:** biological activities, biological screening, *Eunicella cavolini*, gorgonian corals, organic extracts

## Abstract

Due to sedentary lifestyle and harsh environmental conditions, gorgonian coral extracts are recognized as a rich source of novel compounds with various biological activities, of interest to the pharmaceutical and cosmetic industries. The presented study aimed to perform chemical screening of organic extracts and semi-purified fractions obtained from the common Adriatic gorgonian, sea fan, *Eunicella cavolini* (Koch, 1887) and explore its abilities to exert different biological effects in vitro. Qualitative chemical evaluation revealed the presence of several classes of secondary metabolites extended with mass spectrometry analysis and tentative dereplication by using Global Natural Product Social Molecular Networking online platform (GNPS). Furthermore, fractions **F4** and **F3** showed the highest phenolic (3.28 ± 0.04 mg GAE/g sample) and carotene (23.11 ± 2.48 mg β-CA/g sample) content, respectively. The fraction **F3** inhibited 50% of DPPH (2,2-diphenyl-1-picryl-hydrazyl-hydrate) and ABTS (2,2′-azino-bis (3-ethylbenzthiazolin-6-yl) sulfonic acid) radicals at the concentrations of 767.09 ± 11.57 and 157.16 ± 10.83 µg/mL, respectively. The highest anti-inflammatory potential was exhibited by **F2** (IC_50_ = 198.70 ± 28.77 µg/mL) regarding the inhibition of albumin denaturation and **F1** (IC_50_ = 254.49 ± 49.17 µg/mL) in terms of soybean lipoxygenase inhibition. In addition, the most pronounced antiproliferative effects were observed for all samples (IC_50_ ranging from 0.82 ± 0.14–231.18 ± 46.13 µg/mL) against several carcinoma cell lines, but also towards non-transformed human fibroblasts pointing to a generally cytotoxic effect. In addition, the antibacterial activity was tested by broth microdilution assay against three human pathogenic bacteria: *Escherichia coli*, *Pseudomonas aeruginosa*, and *Staphylococcus aureus*. The latter was the most affected by fractions **F2** and **F3**. Finally, further purification, isolation and characterization of pure compounds from the most active fractions are under investigation.

## 1. Introduction

Due to the extreme conditions (temperature, salinity, pressure, variable illumination) of harsh marine environment, a constant need for the protection from predators and to aid in the hunting process as well as to defend their territory against invading competitors, the flora and fauna of the marine ecosystem are known for their broad chemo- and biodiversity [1,2]. Marine organisms have been also used for centuries as food and as traditional medicines for the treatment of various diseases, mainly by Asiatic nations who have recognized their health benefits and ethnopharmacological potential [3,4]. However, only in the last century, marine species have started to be more studied as new resources of bioactive compounds [5,6,7]. Various secondary metabolites, including alkaloids, sterols, terpenoids, peptides and polyketides with unique structural motives and diverse biological activities have been isolated from both marine invertebrates and vertebrates [6]. Consequently, many biologically active marine natural products have been reported in the scientific literature [8,9,10,11,12,13,14,15]. In particular, sponges and their symbiotic microorganisms were identified as the richest source of secondary metabolites with broad pallet of biological activities. On the other hand, cnidarians have been significantly less researched [5,16,17,18]. This particularly refers to sedentary soft and stony coral organisms that produce secondary metabolites with protective or signaling functions in order to survive in their often very specific and harsh marine habitats [5,19,20].

*Eunicella* species, mainly known for their branched growth, belong to the gorgonian soft corals [21,22,23]. Gorgonians are often considered as the ‘sea forests’ due to morphological features and function contributing to their important ecological role on the hard or soft sea bottoms and as such, significantly contribute to the biodiversity of Mediterranean Sea basin [19,24]. Recently, several population studies have revealed that climate changes accelerated by anthropogenic impact, have led to numerous mass mortality events of *Eunicella* coral assemblages [25,26,27]. These circumstances along with the slow growth and recovery pattern, reveal a need for systematic scientific studies on their adaptation to the new ecological conditions [21]. Yellow-orange *E. cavolini* (Koch, 1887) and white *E. singularis* (Esper, 1791) differ in the depth of their habitats and are two species of *Eunicella* genus that currently dominate the coral communities of the Mediterranean Sea [21,28]. Secondary metabolites and their biological effects of *Eunicella* species have only very recently been covered by the scientific literature. A number of metabolites isolated from these corals, were shown to exhibit high cytotoxic, antimicrobial, and anti-inflammatory activities [29]. As there is an imperative need for novel anticancer drugs with new modes of action, significant research has been performed for the identification of new leads derived from natural products [8]. However, despite enormous efforts and improvements in oncology treatments, cancer has been identified among the leading health problems and causes of death, worldwide. Over recent decades, marine organisms were finally recognized as a new and rich source of bioactive natural products that might serve as scaffolds for the production of semisynthetic and synthetic derivatives to be turned into drugs and investigated in clinical trials [30].

With these assumptions in mind and within our platform of the Adriatic Sea bioprospecting programme [31], we evaluated chemical composition and biological activities of the yellow gorgonian, *E. cavolini* from the Adriatic Sea. Herein, we present results of antiproliferative, antibacterial, antioxidative and anti-inflammatory activities screenings of organic extracts and corresponding, semi-purified fractions of *E. cavolini*.

## 2. Results and Discussion

*Eunicella cavolini* belongs to the order of Alcyonacea, phylum Cnidaria that comprises of more than 11,000 species [32]. As reviewed by Rocha et al. [5] and our group [29], gorgonians have been already proven as a rich source of secondary metabolites displaying various biological activities. The isolated bioactive compounds were mostly terpenoids and steroids with antitumor, anti-inflammatory, antimicrobial, antimalarial and antifoulant properties [5]. Herein, the qualitative and quantitative chemical analyses of the extract and semi-purified fractions of *E. cavolini* were performed. Furthermore, their antioxidative, antiproliferative, antibacterial and anti-inflammatory activities were examined.

### 2.1. Extraction Yield, Qualitative and Quantitative Chemical Screening

The exhaustive maceration of 492 g of *E*. *cavolini* resulted in 6.81 g of organic extract (**OE**), representing an extraction yield of 1.38%. Further purification of **OE** using three different organic solvents (cyclohexane, ethyl acetate and methanol) in a certain ratio yielded four semi-purified fractions of distinct polarity with the overall yields being 0.73, 0.13, 0.13 and 0.24% for **F1**–**F4**, respectively (Table 1). Although the extraction optimization was not the main purpose of this research, the obtained yield was lower compared to the studies of Ioannou et al. [33,34,35]. It is possible that different extraction time, sample preparation or the health status of coral colony, particularly overgrowth, necrosis and mass mortality events hitting gorgonians lately, could contribute to the different growth and biochemical composition [26,27,36,37]. The latter might be also affected by different area of sampling as already suggested by Leal et al. [18].

In order to examine the metabolomic profiles of **OE** and **F1**–**F4** samples, they were subjected to qualitative chemical screening based on colorimetric reactions which revealed the presence of several classes of metabolites (Table 2). The observed results confirmed the presence of saponins, glycosides and phenols for the first time in *E. cavolini* samples in addition to the alkaloids [38], steroids [33,34,35,39] and terpenoids [40,41] which were already isolated by Greek and French-Italian scientific groups (Table 2). Alkaloids, terpenoids, phenols and glycosides were present in all samples. However, only fractions **F3** and **F4** contained saponins, while sterols could not be detected in fraction **F4**. Phytochemical tests are not specific for individual classes of compounds. For instance, the Keller-Killiani test is used to detect cardiac glycosides whereas the aglycone moiety can be present due to other types of secondary metabolites. The presence of sterols and terpenes correlates well with the fact that up to 90% of organic extract of soft corals contains sterols, while the rest being terpenes as reported by Edrada et al. [42]. Bracco et al. [43] pointed out that carotenes and derivatives such as xanthophylls are the most abundant pigments in animals as they are substantial in the process of photosynthesis and responsible for the color of coral tissues. Nevertheless, the presence of the herein detected saponins, glycosides and phenols might also be of great interest because of their potential pharmacological utilization, with emphasis on anticancer activity [44,45,46].

In addition, both, total phenolic and carotene contents (TPC and TCC, respectively) were confirmed in all samples followed by quantitative experiments (Table 1). Total phenolic content was determined by a Folin-Ciocalteu (FC) assay and expressed as gallic acid equivalents (GAE). It was the highest in **F4** (3.28 ± 0.04 mg GAE/g sample) while **F1** had the lowest content (1.05 ± 0.09 mg GAE/g sample). This might be in accordance with the highest polarity of the eluent system used to obtain the semi-purified fractions. On the contrary, total phenols were also detected in *E. singularis* at much higher content (>30 mg GAE/g sample), however, even more polar solvents were used for extraction that may result in higher recovery of phenolic compounds from natural matrices [47]. On the other hand, according to Prior et al. [48], FC reagent can react with non-phenolic metabolites as well, including alkaloids (i.e., nucleosides) which were also detected in the samples. Alkaloid compounds may therefore contribute to the overall phenolic content and thus, only rough estimation of their content may be achieved [48]

Although carotenoids, such as astaxanthin and canthaxanthin were isolated from gorgonian coral, *Corallium rubrum*, [43,49] their presence in *Eunicella* species was not yet studied. Therefore, the total carotene content of *E. cavolini* was analyzed as a preliminary examination of the presence of terpenoids. TCC, given as β-carotene equivalent, was the highest in **F3** (23.11 ± 2.48 mg β-CA/g sample) and the lowest in **OE** (4.93 ± 0.05 mg β-CA/g sample) (Table 1). However, these results need to be interpreted carefully since the same experimental setup was used as for qualitatively spectrophotometrically determination of terpenoids and thus, the affection of the overall results by the other types of terpenes cannot be excluded.

Qualitative chemical screening was expanded by mass spectrometry analysis of **OE**. MS/MS data were used to perform tentative compounds dereplication by matching experimental spectra to spectral libraries by use of the Global Natural Product Social Molecular Networking (GNPS) online platform. The best spectral matches (Figures S6–S20) and estimated compounds are presented in Table 3. Such molecular analysis allowed for identification of 15 tentative compounds classified either as terpenoids, steroids, alkaloids or compounds containing phenolic moiety as observed qualitatively. Additional matching of spectra with other public databases might be useful for more robust compounds identifications.

### 2.2. Antioxidant Activities

Previous studies have demonstrated that gorgonians as well as other soft coral species produce non-enzymatic, free radical scavengers to counteract negative effects generated during inflammatory responses [50,51,52,53]. Furthermore, corals exhibit higher antioxidant potential during temperature deviations associated with environmental changes [54,55]. Nevertheless, the antioxidant activity of soft and gorgonian corals have been scarcely examined [50,56].

In present study, antioxidant activity of **OE** and its semi-purified fractions (**F1**–**F4**) was evaluated through inhibition of DPPH and ABTS radicals based on electron-transfer. As shown in Table 4, **F3** displayed the highest antioxidant potential with IC_50_ values of 767.09 ± 11.57 and 157.16 ± 10.83 µg/mL against DPPH and ABTS radicals, respectively. In contrast, **F4** failed to exhibit IC_50_ values in both assays in the tested concentration range. It is important to note that none of the tested samples has displayed potent scavenging activity compared to Trolox used as a positive standard. Interestingly, studies on *E. singularis* performed by Deghrigue et al. [47,57] showed that more polar fractions had higher DPPH radical scavenging potential as a result of higher phenolic content. In our case, it may be presumed that other metabolites contribute more to observed biological activity. This is supported by the carotene content being the highest in the most active fraction, **F3** and generally high antioxidant activities of carotenoids (i.e., astaxanthin) [58,59].

A difference observed between those two assays, might occurred due to the wavelengths used to measure the radical inhibition, namely 550 and 734 nm for DPPH and ABTS radicals, respectively. The latter assay on longer wavelength was used since the first overlaps and interfere with the absorbance values of extract/fractions resulting in a more difficult interpretation of the results. The observed interference can be eliminated by subtraction of the absorbance of the pure sample, however, the resulting curve might not show the real situation as discussed in detail by Apak et al. [60]. Furthermore, DPPH is a hindered, stable and long-lived radical whose inhibition is slower than the corresponding inhibition of ABTS radical, showing other disadvantages such as steric effects, more complex chemistry of redox reactions and the presence of contaminants [60,61,62].

### 2.3. In Vitro Anti-Inflammatory Activities

Nowadays, the discovery of novel agents as substitutes for non-steroidal anti-inflammatory drugs whose administration often leads to harmful side effects is highly needed [63]. To the best of our knowledge, only *E. singularis* seems to have been screened for anti-inflammatory activity by Deghrigue et al. [63,64] who observed an inhibition of carrageenan-induced oedema in rats. In our experiment, two in vitro assays for this activity evaluation were used, the inhibition of albumin denaturation and the inhibition of soybean lipoxygenase. The latter is a well described essential enzyme responsible for the synthesis of inflammation mediators (leukotrienes) involved in several inflammatory processes [65]. Denaturation of proteins may be correlated with inflammation processes such as arthritis making the first assay a screening tool for anti-arthritic activity [66]. As discussed by Williams et al. [67], the major advantages of abovementioned assays is the removal of additional animal experiments, particularly when working with crude extracts or low amounts of samples to conduct the studies. Both assays are rarely employed in the studies of marine-derived extracts or compounds, but tested on marine sponge sample of *Hyrtios erectus* [68].

As summarized in Table 5, **F2** exhibited the highest inhibitory potential against albumin denaturation (IC_50_ = 198.70 ± 28.77 µg/mL). On the other hand, at a concentration of 254.49 ± 49.17 µg/mL, **F1** reduced the activity of lipoxygenase by 50% while **OE** displayed the highest IC_50_ value (812.19 ± 85.99 µg/mL). Further, **F4** did not cause 50% of inhibition in either of the assays. However, the IC_50_ values of both **F2** and **F1** in terms of albumin denaturation and lipoxygenase inhibition, respectively were still higher than of indomethacin which anti-inflammatory activity is consistent with previous results obtained by Bouhlali et al. [69] and Sircar et al. [70].

The observed anti-inflammatory activity for the samples of *E. cavolini* may be attributed to the presence of steroids and terpenoids which were extensively reviewed for such activity [5,71,72,73,74]. Eunicellin-type diterpenoids demonstrated the ability of inhibition of inducible nitric oxide synthase (iNOS) and cyclooxygenase (COX-2) proteins in macrophages after stimulation by lipopolysaccharide (LPS) [73]. Furthermore, alkaloids of marine origin which exhibited anti-inflammatory activity have just recently been discussed by Souza et al. [74]. In addition, it was assumed that due to lipophilicity, fraction **F1** may contain less polar compounds (fatty acids) which might be present in *E. cavolini* samples as obtained by GNPS online platform and which could compete and inhibit the same active site as a substrate, linoleic acid [75].

### 2.4. Evaluation of Antiproliferative Activity

Many studies regarding the biological activities of marine natural compounds produced by cnidarians were conducted in the field of cancer research [5]. Indeed, according to Rocha et al. [5], more than 40% of isolated bioactives were found to possess potential antitumor activity. In accordance with this observation, antiproliferative and antitumor activities of extracts and pure compounds of *Eunicella* species were the major subjects of numerous studies [29].

Accordingly, the antiproliferative effects of *E. cavolini* extracts was determined by means of colorimetric MTT assay against four human tumour cell lines (colorectal, pancreatic and breast adenocarcinoma and hepatocellular carcinoma) and one non-transformed line (human fibroblasts). The data, summarized in Table 6, indicate moderate to high inhibitory activity which was generally non-selective towards specific tested cell lines. Fractions **F2** and **F3** exhibited the highest activity against SW620, CFPAC-1, MCF-7 and HepG2 cells with IC_50_ values: 0.82 ± 0.14, 5.61 ± 2.11, 5.21 ± 1.03 and 2.58 ± 0.57 µg/mL, respectively for **F2**. The activity of **F2** against SW620 cells was comparable to 5-fluorouracil (5-FU) while the determined IC_50_ value against HepG2 was lower than the standard drug. Moreover, 5-FU displayed higher activity towards the remaining cell lines. At concentrations of 5.73 ± 2.97, 7.93 ± 0.12, 14.63 ± 9.41 and 6.48 ± 1.91 µg/mL, **F3** inhibited 50% growth of tested tumour cell lines, respectively. Further on, **F1** was the least active against SW620 and HepG2 cells when compared to the rest of tested samples. Large deviations in some cases could be attributed to the low level of cell cycle synchronization, which is associated with the possibility that cell cycle phase has an effect on the sensitivity of cancer cells to the test compound or extracts [76].

For the first time, the effect of extract and semi-purified fractions on normal, non-transformed human cell line HFF-1 was examined. In that case, all samples, except **F1**, strongly inhibited the proliferation of non-transformed cells (HFF-1) at a concentration less than 10 µg/mL. Furthermore, a selectivity index (SI) was determined emphasizing that an SI < 2 points to general toxic effect (Table 7) [77,78].

Calculated SI was about or less than 1 for **OE**, **F2**, **F3** and **F4**, while the highest for **F1** against CFPAC-1 (2.85) and MCF-7 (3.10) cells (Table 7). Therefore, **F1** was more selective towards CFPAC-1 and MCF-7, although it also showed moderate activity against normal cells. Finally, 5-FU showed 4 times greater selectivity towards MCF-7 cells in regard to **F1**.

As hypothesized by Mancini et al. [40], diterpenoids might influence the selectivity in inhibition of cancer cells. The most pronounced antiproliferative effects attributed to **F2** and **F3** can be explained by the presence of sterols and terpenoids, that were already extracted from *Eunicella* corals and assayed against various cancer cell lines [29,33,34,35]. However, since this class of compounds were found in other fractions and organic extract, it remains to be determined whether the amount of each individual class of compounds present in a particular sample contributes to the observed differences in antiproliferative activity. Since **F3** demonstrated the highest radical-scavenging and antiproliferative activity, associated with the highest carotene content, we assume that those chemicals might contribute to observed effect as well, taking into consideration known anticancer effect of carotenoids isolated from seaweeds [79]. The assumption on antiproliferative activity associated with antioxidant potential was also reported by Deghrigue et al. [57].

### 2.5. Evaluation of Antibacterial Activity

To date, many studies have been reported regarding antimicrobial activity of gorgonian corals’ extract or pure compounds [80,81,82,83,84,85]. The antibacterial activity was evaluated by broth microdilution assay against three human pathogens: *Escherichia coli*, *Pseudomonas aeruginosa*, and *Staphylococcus aureus*. The results demonstrated partial selective inhibition of tested bacterial species, as summarized in Table 8 with the highest activity against *S. aureus*. The same strain was also inhibited by epidioxysterol which can be found in *Eunicella* species as published by Liang et al. [86]. Both, **F2** and **F3** displayed comparable EC_50_ values against *S. aureus*, being 175 ± 27 and 171 ± 68 µg/mL, respectively. The growth of *E. coli* was inhibited the most by **F2** (255 ± 199 µg/mL). Interestingly, only **F1** fraction exhibited EC_50_ value of 523 ± 48 µg/mL after incubation with *P. aeruginosa*, making it the least susceptible to the tested samples of *E. cavolini*. Importantly, the organic extract **OE** and fractions **F1**–**F4** of *E. cavolini* had lower antibacterial activity comparing to the positive control, chloramphenicol, which EC_50_ values were measured as 0.75 ± 0.09 and 1.50 ± 0.10 µg/mL against *E. coli* and *S. aureus*, respectively. Since inhibition of growth by **F1** is relatively similar for all three bacterial species, it could indicate for instance, the presence of substances that are active on both Gram negative and Gram positive species (though not in a sufficient quantity to produce complete inhibition) or there is an interaction between some compounds that exert such an effect, yet missing in other fractions. Further bioassay-guided fractionations and retesting will hopefully provide clues to this effect. Importantly, the relatively large standard deviation might arise from of large variability at higher concentrations of all samples. Another explanation might lie in possible interactions within samples (synergisms and antagonisms) or in different effects (stimulation or inhibition) of various compounds presented in the sample without any interaction of the two.

Please note that *E. coli* and *S. aureus* in our assays had minimum inhibitory concentrations (MIC) of 4 and 8 μg/mL for chloramphenicol, respectively, and are used as quality controls of the assays as per Clinical & Laboratory Standards Institute (CLSI) guidelines for the assessment of validity of the results obtained. Only fractions **F2** and **F3** have provided us with MIC values at the highest tested concentration of 2500 µg/mL (*w*/*v*) which was only observed with *S. aureus* and not the other two bacterial species. Therefore, given results could indicate the potential antibacterial activity.

Partial inhibition of our samples agrees with the hypothesis made by Jensen et al. [83]. The authors assumed that lower activity can be explained by both, structure of bacteria and existence of symbiotic communities. Bacterial community plays an important role in coral functioning, providing nitrogen fixation, food supply and antibiotic production. For that matter, bacteria of the genus *Endozoicomonas* are the most abundant in symbiotic association with *E. cavolini* [87]. Considering mortality events and temperature rise, Bally and Garrabou [88] isolated a *Vibrio coralliilyticus* strain from diseased specimens of the red gorgonian, *Paramuricea clavata*, indicating the importance of pathogenic bacteria, as well. Finally, some bacteria have been related to fouling processes which eventually leads to colonial death [89]. Therefore, it could be concluded that corals maintain balance in inhibiting pathogen bacteria and producing compounds of lower activity against beneficial bacteria.

On the other hand, our bacterial panel consisted only of human pathogenic bacteria, none of which is considered a marine species. A study of microbial regulation in Caribbean gorgonian corals reported the antimicrobial activity of ethanol extracts being significantly higher against non-marine than marine bacteria and also significantly higher against Gram positive than Gram negative bacteria [90]. The latter was also observed in our study since Gram positive *S. aureus* was more affected by *E. cavolini* samples, however we cannot fully claim that Gram positive bacteria are more sensitive to the inhibitory activity of the samples due to a limited bacterial panel used in our experiment. Therefore, in order to fully prove and elucidate these associations, further investigation is required including also marine bacteria.

Finally, the highest activity of **F3** and **F2** is consistent with the correlation between antioxidant and antimicrobial activities which were reviewed by Shannon et al. [91], emphasizing carotenoids and polyphenols as antibacterial metabolites and their potential action of mechanism [92].

**Figure 1:**
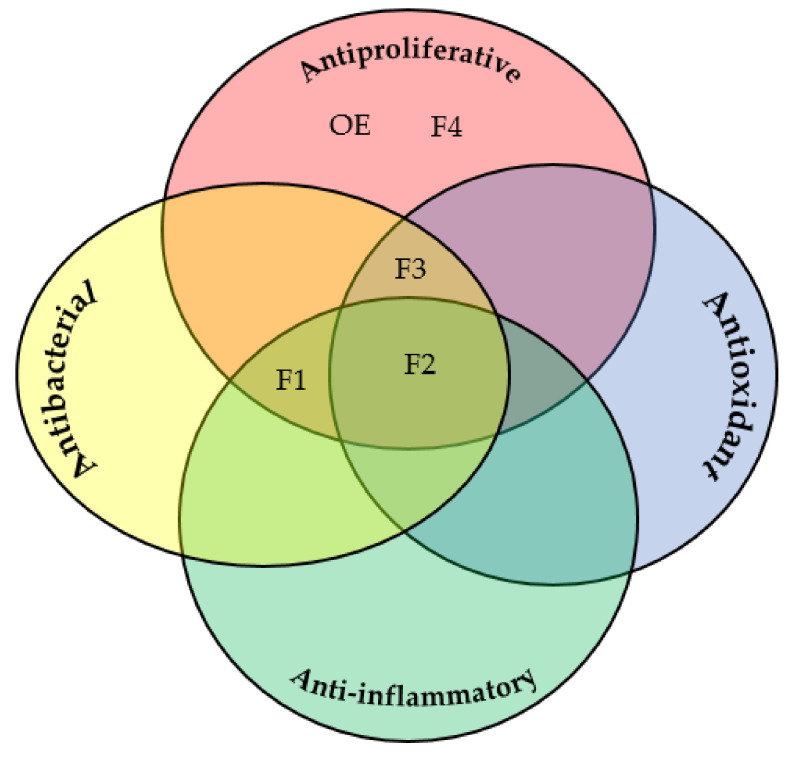
Observed biological activities for **OE** and **F1**–**F4** obtained from *E. cavolini.*

In summary, the herein tested extracts and semi-purified fractions of the Adriatic Sea gorgonian, *E. cavolini* exhibited interesting biological activities (Figure 1) with differences among them that may arise from divergence in concentrations and type of extracted bioactive secondary metabolites. Furthermore, the variation of biological activities of *Eunicella* species reported herein and those available in the literature might be attributed to the different geographic location of sampling and sampling season [93], as well as the presence or absence of certain microorganisms such as zooxanthellae or bacteria [22]. The latter hypothesis is consistent with increasing evidence of microorganisms being producers of the metabolites isolated from coral animals [5,56]. Therefore, a metagenomic procedure [94] needs to be performed in order to certainly conclude whether all metabolites mentioned above and, consequently, responsible for observed biological activities are being synthesized by only coral or coral-associated symbiotic microorganisms [87]. Metagenomic analysis is accordingly underway and will eventually provide new insights into bioprospecting of *E. cavolini* accompanied with bioassay-guided comprehensive fractionation of obtained organic extracts.

## 3. Materials and Methods

### 3.1. Materials

Acetic anhydride, acetic acid, sulphuric acid (96%), iron(III) chloride, potassium iodide and mercury(II) chloride were purchased from Kemika (Zagreb, Croatia). Methanol, dichloromethane, ethyl acetate, cyclohexane and potassium peroxodisulphate were obtained from VWR Chemicals (Radnor, PA, USA) while chloroform was obtained from Honeywell (Charlotte, NC, USA). ABTS and linoleic acid were purchased from Alfa Aesar (Kandel, Germany). Trolox^®^ was obtained from Acros Organics (Geel, Belgium). DPPH, β-carotene and lipoxidase type I-B from soybean were purchased from Sigma-Aldrich (Darmstadt, Germany). Finally, Dulbecco’s modified Eagle medium (DMEM), foetal bovine serum (FBS), L-glutamine, penicillin and streptomycin were obtained from Capricorn Scientific (Ebsdorfergrund, Germany).

### 3.2. Collection and Extraction of Animal Material

The yellow gorgonian, *Eunicella cavolini*, was collected from the Adriatic Sea, near the Island of Pag (Paška vrata) in May 2018 at a depth between 20 and 30 m and kept at −80 °C. Fresh organism was washed, dried, weighed, cut into small pieces and macerated for 24 h at room temperature by using dichloromethane and methanol (3:1, *v*/*v*, 3 L) to obtain 6.81 g (1.38%) of dark red-orange oily organic extract **OE** after evaporation of solvent under reduced pressure at 40 °C, while the light yellow aqueous layer (pH = 8.20) was discarded leaving the organic part. The organic extract **OE** was further purified by gravity column chromatography on silica gel to afford 4 semi-purified fractions by using different solvent systems as the mobile phase of increased polarity: c-hexane/ethyl acetate (3:1, *v*/*v*, **F1**), c-hexane/ethyl acetate (1:1, *v*/*v*, **F2**), ethyl acetate (**F3**) and ethyl acetate/methanol (4:1, *v*/*v*, **F4**). Samples of **OE** and **F1**–**F4** were concentrated, documented by mass spectrometry and NMR, freeze-dried and kept at −20 °C for further biological and chemical analyses.

### 3.3. Qualitative Chemical Screening

The presence of secondary bioactive metabolites in both, the extract and fractions was evaluated by specific colorimetric tests: phenolic compounds (ferric chloride test), terpenoids (Salkowski test), alkaloids (Mayer’s test), glycosides (Keller-Killiani test), steroids (Libermann-Burchard’s test) and saponins (froth test) according to Edeoga et al., followed by minor modifications [95]. All standard solutions were prepared by dissolving dried extracts or semi-purified fractions in methanol at a concentration of 2 mg/mL.

(a) Test for terpenoids (Salkowski test):

Standard solution (2 mL) was mixed with chloroform (2 mL) in a test tube. This was followed by the addition of concentrated H_2_SO_4_ (2 mL, 96% aq), dropwise along the side of test tube to form a layer. The formation of red-brown coloration of the interface indicates the presence of terpenoids.

(b) Test for saponins (froth test):

Standard solution (2 mL) was diluted with deionized water (2 mL) and vigorously shaken for few minutes. A formation of stable foam on the top of the mixture indicates the presence of saponins.

(c) Test for sterols (Liebermann-Burchard’s test):

Standard solution (1 mL) was diluted by acetic acid anhydride (2 mL) and chloroform (1 mL) were added. Few drops of concentrated H_2_SO_4_ were then added dropwise along the side of test tube. The color change from violet to blue or green indicates the presence of sterols.

(d) Test for alkaloids (Mayer’s test):

Standard solution (2 mL) was mixed with of Mayer’s reagent (2 mL) which was prepared by dissolving of mercury(II) chloride (1.36 g, 5.01 mmol) and potassium iodide (5 g, 30.12 mmol) in deionized water (100 mL). Formation of color cream precipitate indicates the presence of alkaloids.

(e) Test for phenols (Folin-Ciocalteu’s test):

Standard solution (100 µL) was mixed with Na_2_CO_3_ (2 mL, 2% aq) and left for few minutes at room temperature which was followed by the addition of Folin-Ciocalteu’s reagent (100 µL, 50% aq). The mixture was incubated for 30 min at room temperature in the dark and observed for appearance of blue color which indicates the presence of phenols.

(f) Test for glycosides (Keller-Killiani test):

Standard solution (2 mL) was treated with glacial acetic acid (2 mL) which contained FeCl_3_ (2 drops, 5% aq). This was followed by the addition of conc. H_2_SO_4_ (400 µL), dropwise along the side of test tube. Formation of red-brown ring of the interface indicates the presence of glycosides with a violet ring which may also appear below the brown ring.

### 3.4. Total Phenolic Content and Total Carotene Content

(a) Total phenolic content (TPC)

TPCs of OE and **F1**–**F4** fractions were estimated by the method of Taga et al. [96] 100 µL of sample extract was mixed with Na_2_CO_3_ (2 mL, 2% aq) and left for few minutes at room temperature which was followed by the addition of Folin-Ciocalteu’s reagent (100 µL, 50% aq). The mixture was incubated for 30 min at room temperature in the dark and the absorbance of solution was measured at 720 nm. A calibration curve of gallic acid (GA) was obtained for concentration range: 1000–62.5 µg/mL and the total phenolic content was calculated using formula:

TPC = (c × V)/m,(1)

where TPC is total phenolic content expressed as mg of GA/g of sample, c is concentration of gallic acid obtained from calibration curve, V is volume of sample and m is the mass of sample.

(b) Total carotene content (TCC)

Total tetraterpenoid (carotene) content (TCC) was estimated by colorimetric assay following the procedure of Ghorai et al. [97], similarly as Salkowski test described above (Section 3.3a). In brief, methanolic solution (200 µL) of OE and **F1**–**F4** was mixed with chloroform (1.5 mL) in a test tube. Samples were mixed thoroughly which was followed by addition of conc. H_2_SO_4_ (100 µL) and incubation period of 30 min for red-brown coloration of the interface to appear. Supernatant liquid was then removed, carefully, without disturbing the colored ring. Eventually, methanol (100 µL) was added to dilute the coloration and the samples were transferred to the 96-well plate to read the absorbance at 538 nm. A solution of β-carotene in methanol (concentration range: 1000, 500, 250, 125 and 62.5 µg/mL) was used to prepare a calibration curve, however, the incubation time lasted for 5 min. Finally, total carotene content was calculated using the same formula as for TPC (2.5a).

### 3.5. MS/MS Molecular Networking

A molecular network was created using the online workflow (https://ccms-ucsd.github.io/GNPSDocumentation/) on the GNPS website (http://gnps.ucsd.edu) [98] and MS/MS data which experimental setup is provided in Supplementary. The data was filtered by removing all MS/MS fragment ions within +/− 17 Da of the precursor *m/z*. MS/MS spectra were window filtered by choosing only the top 6 fragment ions in the +/− 50Da window throughout the spectrum. The precursor ion mass tolerance was set to 2.0 Da and a MS/MS fragment ion tolerance of 0.9 Da. A network was then created where edges were filtered to have a cosine score above 0.8 and more than 6 matched peaks. Further, edges between two nodes were kept in the network if and only if each of the nodes appeared in each other’s respective top 10 most similar nodes. Finally, the maximum size of a molecular family was set to 0, and the lowest scoring edges were removed from molecular families until the molecular family size was below this threshold. The spectra in the network were then searched against GNPS’ spectral libraries. The library spectra were filtered in the same manner as the input data. All matches kept between network spectra and library spectra were required to have a score above 0.8 and at least 8 matched peaks.

### 3.6. Assessment of Antioxidant Activity

(a) DPPH radical-scavenging activity:

The in vitro antioxidant activities of OE and **F1**–**F4** were evaluated by a (2,2-diphenyl-1-picrylhydrazyl) (DPPH)-radical assay according to protocol described by Kim et al. after minor modifications [99]. Briefly, methanolic solution (100 µL) of OE and **F1**–**F4** of various concentrations (final concentrations: 1000, 500, 250, 125 and 62.5 µg/mL) was mixed with DPPH (100 µL, final concentration: 0.01 mM) in a 96-well plate. The plate was incubated for 30 min in the dark and the absorbance was read at 550 nm against the blank (methanol) using microplate reader. The same amount of methanol in DPPH solution was used as a negative control. Trolox (6-hydroxy-2,5,7,8-tetramethylchroman-2-carboxylic acid) at the same concentration range as *E*. *cavolini* samples, mentioned above, was used as a standard. The radical scavenging activity was expressed as percentage inhibition of DPPH and calculated according to the formula:

% inhibition = 100 × (1 − A_S_/A_C_) (2)

where A_C_ and A_S_ stand for the absorbances of the control and sample, respectively. The concentration resulting in 50% inhibition of DPPH radical (IC_50_) was determined from the graph which presents the inhibition percentage plotted against the concentration of the samples. All measurements were performed in three independent experiments.

(b) ABTS radical-scavenging activity:

The in vitro antioxidant activity of the OE and **F1**–**F4** fractions was also investigated by a 2,2′-azino-bis(3-ethylbenzothiazoline-6-sulfonic acid diammonium salt (ABTS)-radical assay according to study performed by Zheleva-Dimitrova et al. [100] with some minor modifications. Firstly, the ABTS radical was prepared by mixing of the equimolar amount of ABTS solution (7 mM) and potassium persulfate solution (2.4 mM) which was incubated in the dark overnight at room temperature. The obtained solution was diluted with methanol and the absorbance at 734 nm was measured as 0.700. The solutions of samples (100 µL) were prepared in methanol (final concentration in range: 1000–62.5 µg/mL) and were then mixed with ABTS-solution (100 µL) in a 96-well plate. The plate was incubated for 5 min in the dark and the absorbance was measured at 734 nm against the blank (methanol) using microplate reader. The ABTS-solution diluted by the same amount of methanol (100 µL) was used as a negative control. Radical scavenging activity was calculated according to the formula mentioned for DPPH assay (*vide supra*). Antioxidant activity of *E*. *cavolini* samples was compared to Trolox at the same concentration range which was used as a positive control. IC_50_ value, which denotes the concentration resulting in 50% inhibition of ABTS radical was determined from the graph which presents the inhibition percentage plotted against the concentration of the samples. All measurements were performed in three independent experiments.

### 3.7. Assessment of In Vitro Anti-Inflammatory Activity

(a) Inhibition of albumin denaturation

The inhibitory activity of OE and **F1**–**F4** against albumin denaturation was tested by the method described by Williams et al. [67] with modifications of volume followed after optimization procedures. A reaction vessel consisted of 500 µL of bovine serum albumin (1% in PBS, pH 6.4) and the same amount of tested sample with final concentration range: 500, 250, 125, 62.5 and 31.3 µg/mL). Deionized water (500 µL) instead of tested samples was used as a negative control which indicates 100% of denaturation. The mixtures were incubated at 37 °C for 15 min after which heating at 90 °C for 3 min followed in order to cause albumin denaturation. After cooling, the turbidity of the samples was measured at 660 nm. The percentage of albumin denaturation inhibition was calculated following the formula:

% inhibition = 100 × (1 − A_S_/A_C_) (3)

where A_C_ and A_S_ stand for the absorbances of the control and sample, respectively. The concentration resulting in 50% inhibition of albumin denaturation (IC_50_) was determined from the graph which presents the inhibition percentage plotted against the concentration of the samples. Indomethacin was used in the same concentration range as mentioned above as a positive control and for comparison.

(b) Inhibition of soybean lipoxygenase (sLOX)

The inhibitory activity of methanolic samples of OE and **F1**–**F4** against sLOX was investigated using linoleic acid as a substrate, applying the protocol by Oso et al. with modification regarding the final volume of each component [101]. In short, the sample (50 µL, concentration range: 1000, 500, 250, 125 and 62.5 µg/mL) was mixed with PBS (50 µL, pH = 8.52). Addition of sLOX (30 µL, final concentration units: 1300 U/mL) enzyme solution followed with incubation time of 5 min at 35 °C. Afterwards, 100 µL of linoleic acid (0.26 mM) was added and mixed gently for 10 min at the same temperature. For negative control, PBS (50 µL) instead of samples was added, indicating 100% of sLOX activity. The absorbance was read at 234 nm. The percentage of lipoxygenase inhibition was calculated following the formula:

% inhibition = 100 × (1 − A_S_/A_C_) (4)

where A_C_ and A_S_ stand for the absorbances of the control and sample, respectively. The concentration resulting in 50% inhibition of sLOX activity (IC_50_) was determined from the graph which presents the inhibition percentage plotted against the concentration of the samples. Indomethacin was used as a positive control and for comparison (final concentration range: 250, 125, 62.5, 31.3 and 15.6 µg/mL).

### 3.8. Evaluation of In Vitro Antiproliferative Effect

The human carcinoma cell lines colorectal adenocarcinoma (SW620), ductal pancreatic adenocarcinoma, metastatic breast cancer (MCF7), hepatocellular carcinoma as well as normal skin fibroblasts (HFF) obtained from the American Type Culture Collection (ATCC, Manassas, VA, USA), were cultured in Dulbecco’s modified Eagle medium (DMEM) supplemented with foetal bovine serum (FBS, 10% aq.), L-glutamine (2 mM), penicillin (100 U/mL) and streptomycin (100 µg/mL) in a humidified atmosphere with 5% CO_2_ at 37 °C in humidified atmosphere. Lyophilized extracts were then dissolved in growth medium containing 10% DMSO and further dissolved using vortexing and ultrasonic bath, 30 min at 37 °C. Carcinoma cell lines and normal human fibroblasts were seeded in 96 well plates at density of 3000 cells per well or 5000 cells per well, respectively according to the doubling time of transformed vs. normal cells lines. Prior to the experiment, test extracts were diluted in, five, 10-fold dilutions in growth medium ranging from 10^−4^ to 1 mg/mL. Cells were incubated with test extracts for 72 h while 5-fluorouracil (5-FU) was used as a positive control. Cell growth rate was evaluated using MTT assay according to the manufacturer’s guidelines. Using the formulas proposed by the National Institutes of Health (NIH, Bethesda, MD, USA), the percentage of cell growth was calculated by transforming the experimentally determined absorbance values [102]. Concentrations of extracts needed to inhibit 50% of cell growth (IC_50_ values) were calculated from dose response curves using linear regression analysis. The selectivity index (SI), as a measure of cytotoxic selectivity of *E*. *cavolini* extract and fractions against cancer cell lines, was calculated according to the following formula [78]:

SI = IC_50_ (non-transformed cell line)/IC_50_(cancer cell line) (5)

### 3.9. Evaluation of Antibacterial Activity

The antibacterial activity of extracts and semi-purified fractions was evaluated by broth microdilution assay according to the Clinical & Laboratory Standards Institute guidelines [103] using *Escherichia coli* NCTC 12241, *Staphylococcus aureus* ATCC 6538, and *Pseudomonas aeruginosa* NCTC 12903 as indicator strains. All assays employed positive (inoculated media without the tested sample) and negative (sterile media) controls and a quality control using chloramphenicol. All assays were performed in duplicates at 35 °C aerobically. The concentration range tested was 5–2000 μg/mL (*w*/*v*) for OE, F2–F4 and 2–1000 μg/mL (*w*/*v*) for F1. Results were interpreted both visually and spectrophotometrically at wavelength of 600 nm using microplate reader, and minimal inhibitory concentrations, if observed, were confirmed by culture isolation from the wells. Dose response curve analyses were performed in RStudio v.1.1.463 environment for R software (R Core Team (2018). R: a language and environment for statistical computing. R Foundation for Statistical Computing, Vienna, Austria. URL https://www.R-project.org/.) using drc package [104]. Effective concentration 50% (EC_50_), a concentration resulting in 50% inhibition of bacterial growth, estimated from log-logistic three parameter dose response models, was used as a comparative feature of relative potency of the tested samples.

## 4. Conclusions

The presented research aimed to analyze chemical properties of extracts and semi-purified fractions of *Eunicella cavolini* collected in the Adriatic Sea, as well as their corresponding potential to exhibit antiproliferative, antioxidant, anti-inflammatory and antibacterial activities. Obtained in vitro data showed that the purification of organic extract resulted in fractions with higher biological activities. In particular, F2 and F3 were found to be more active against tested cancer cell lines (MCF-7, CFPAC-1, HepG2 and SW620) and *Staphylococcus aureus*, which might be correlated with observed potential of radical-scavenging activity and chemicals detected in these samples. Furthermore, the F1 fraction was observed to be a possible inhibitor of lipoxygenase. Our results indicate a diversity of extract/fractions in the type of compounds that may have a synergistic or antagonistic effect. *Eunicella cavolini* extract/fractions may be regarded as a promising source of antiproliferative, antibacterial and anti-inflammatory agents that could be further explored or chemically modified to obtain higher biological activities. The expansion of this study is already on the way and focused on individual identification of extracted secondary metabolites responsible for herein presented results.

## Figures and Tables

**Table 1:** Solvents used for preparation of samples, extraction and purification yields, total phenolic and carotene content of **OE** and **F1**–**F4** obtained from *E*. *cavolini*.

Solvents	Sample Abbreviation	Extraction/ Purification Yield (%) *	TPC (mg GAE/g Sample)	TCC (mg β-CA/g Sample)
**DCM/MeOH = 3/1**	OE	1.38	2.69 ± 0.01	4.93 ± 0.05
***c*-HEX/EA = 3/1**	F1	0.73 (52.86)	1.05 ± 0.09	13.30 ± 1.26
***c*-HEX/EA = 1/1**	F2	0.13 (9.61)	1.21 ± 0.17	16.29 ± 1.48
**EA**	F3	0.13 (9.58)	1.70 ± 0.06	23.11 ± 2.48
**EA/MeOH = 4/1**	F4	0.24 (17.13)	3.28 ± 0.04	10.62 ± 0.97

TPC, total phenolic content; GAE, gallic acid equivalents; TCC, total carotene content; β-CA, β-carotene equivalents. TPC and TCC are expressed as mean value ± standard deviation of triplicate measurement. * Values in parentheses denote extraction yield of fractions from organic extract.

**Table 2:** Qualitative chemical screening of **OE** and **F1**–**F4** obtained from *E*. *cavolini*.

Sample	Saponins	Sterols	Alkaloids	Terpenoids	Glycosides	Phenols
**OE**	−	+	+	+	+	+
**F1**	−	+	+	+	+	+
**F2**	−	+	+	+	+	+
**F3**	+	+	+	+	+	+
**F4**	+	−	+	+	+	+

“+” and “−” indicate the presence or absence of certain class of metabolites, respectively.

**Table 3:** Tentative identification of secondary metabolites from *E. cavolini* organic extract by using GNPS.

	Compound Name	Structure	Class	Exact Mass
1	(2*E*,4*E*)-5-{(1*S*,2*R*,4a*S*,5*S*,8*S*,8a*S*)-2-[(2*E*)-2-Buten-2-yl]-5-hydroxy-3,8-dimethyl-1,2,4a,5,6,7,8,8a-octahydro-1-naphthalenyl}-2,4-pentadienoic acid Phomopsidin)	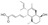	Fatty acyls	330.219
2	(3b*S*,5a*S*,7*R*,8*R*,10a*R*,10b*S*)-3b,4,5,6,7,8,9,10,10a,10b-Decahydro-7-hydroxy-10b-methyl-5a,8-Methano-5aH-cyclohepta(5,6)naphtho(2,1-b)furan-7-methanol (Kahweol)	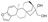	Terpenoids	314.188
3	(5Z,8Z,11Z,14Z)-17,18-Epoxy-5,8,11,14-icosatetraenoic acid	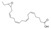	Fatty acids	318.22
4	(2S,4S)-Icos-19-ene-1,2,4-triol	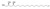	Fatty acyls	328.297
5	5-[2-(Furan-3-yl)ethyl]-8-hydroxy-5,6,8a-trimethyl-3,4,4a,6,7,8-hexahydronaphthalene-1-carboxylic acid *	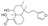	Prenol lipids	332.198
6	3-Hydroxy-4-(2,6,7-trihydroxy-6-methylheptan-2-yl)benzoic acid *	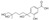	Prenol lipids	298.142
7	(*Z*)-4-Oxo-1-(4-(5-oxotetrahydrofuran-2-yl)butyl)-5-(pent-2-en-1-yl acetate) *	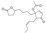	Fatty Acyls	348.194
8	5-[2-(3-Furyl)ethyl]-8a-(hydroxymethyl)-5,6-dimethyl-3,4,4a,5,6,7,8,8a-octahydro-1-naphthalenecarboxylic acid *	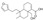	Prenol lipids Diterpenoids	332.199
9	Abieta-8(14),9(11),12-triene-7,18-diol	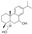	Prenol lipids Diterpenoids	302.225
10	N-[4-[1-[2-(6-methylpyridin-2-yl)ethyl]piperidine-4-carbonyl]phenyl]methanesulfonamide	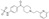	Alkaloids	401.177
11	2’,4’,6’-Trihydroxydihydrochalcone	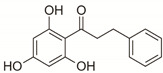	Phenylpropanoids and polyketides	258.089
12	(3*S*,6*R*,6a*S*,7*S*)-6,6a,7,8-Tetrahydroxy-3-methyl-3,4,5,6,7,12a-hexahydro-2*H*-benzo[a]anthracene-1,12-dione	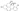	Benzenoids Tetralins	344.125
13	Methyl (1*S*,15*S*,18*S*,19*S*,20*S*)-18-hydroxy-1,3,11,12,14,15,16,17, 18,19,20,21-dodecahydroyohimban-19-carboxylate (Rauwolscine)	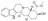	Alkaloids	354.450
14	(9*S*,10*R*,13*R*,14*S*,17*R*)-10,13-dimethyl-17-((*R*)-6-methylheptan-2-yl)-1,2,8,9,10,11,12,13,14,15,16,17-dodecahydro-3*H*-cyclopenta[a]phenanthren-3-one	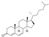	Steroids and steroid derivatives	382.324
15	1-[2,4-Dihydroxy-6-[(2*S*,3*R*,4*S*,5*S*,6*R*)-3,4,5-trihydroxy-6-(hydroxymethyl)oxan-2-yl]oxyphenyl]-3-(4-methoxyphenyl)propan-1-one	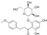	Flavonoid glycosides	450.153

* Complete stereochemistry was not provieded by the GNPS result.

**Table 4:** IC_50_ values of DPPH and ABTS radical-scavenging activity of **OE** and **F1**–**F4** obtained from *E*. *cavolini*. Trolox was used as positive control.

Sample	IC_50_ of DPPH Scavenging Activity (µg/mL)	IC_50_ of ABTS Scavenging Activity (µg/mL)
**OE**	963.67 ± 80.07	395.84 ± 24.25
**F1**	896.22 ± 39.36	>1000
**F2**	984.68 ± 24.35	282.68 ± 23.77
**F3**	767.09 ± 11.57	157.16 ± 10.83
**F4**	>1000	>1000
**Trolox**	<62.5	<62.5

Values are expressed as mean value ± standard deviation of triplicate measurements.

**Table 5:** IC_50_ values of albumin denaturation and soybean lipoxygenase activity of **OE** and **F1**–**F4** obtained from *E. cavolini*. Indomethacin was used as positive control.

Sample	IC_50_ (µg/mL)	
	Inhibition of Albumin Denaturation	Inhibition of Soybean Lipoxygenase
**OE**	319.61 ± 7.85	812.19 ± 85.89
**F1**	331.65 ± 28.30	254.49 ± 49.17
**F2**	198.70 ± 28.77	364.35 ± 23.42
**F3**	458.83 ± 27.99	370.49 ± 17.02
**F4**	>500	>1000
**Indomethacin**	90.33 ± 9.50	34.57 ± 9.12

Values are expressed as mean value ± standard deviation of triplicate measurement.

**Table 6:** In vitro growth inhibitory activity of **OE** and **F1**–**F4** tested against four human cancer cell lines: SW620, CFPAC-1, MCF-7 and HepG2 and non-transformed fibroblast cell line (HFF-1).

Sample	IC_50_ (µg/mL)				
	SW620	CFPAC-1	MCF-7	HepG2	HFF-1
**OE**	13.14 ± 5.02	22.69 ± 4.14	36.20 ± 3.87	30.11 ± 1.44	6.48 ± 0.12
**F1**	158.78 ± 86.47	73.03 ± 14.63	67.01 ± 5.42	231.18 ± 46.13	207.80 ± 21.60
**F2**	0.82 ± 0.14	5.61 ± 2.11	5.21 ± 1.03	2.58 ± 0.57	0.39 ± 0.03
**F3**	5.73 ± 2.97	7.93 ± 0.12	14.63 ± 9.41	6.48 ± 1.91	6.95 ± 1.96
**F4**	18.76 ± 2.61	48.80 ± 1.12	42.76 ± 5.96	37.66 ± 3.47	9.97 ± 1.55
**5-FU**	0.83 ± 0.03	0.84 ± 0.09	0.01 ± 0.001	7.18 ± 1.95	0.12 ± 0.02 *

* Results obtained on lung fibroblast WI38. Values are expressed as mean value ± standard deviation of triplicate measurement.

**Table 7:** Selectivity index (SI) of **OE** and **F1**–**F4** tested against four human cancer cell lines: SW620, CFPAC-1, MCF-7 and HepG2.

Sample	SI			
	SW620	CFPAC-1	MCF-7	HepG2
**OE**	0.49	0.29	0.18	0.22
**F1**	1.3	2.85	3.10	0.90
**F2**	0.48	0.07	0.07	0.15
**F3**	1.21	0.88	0.48	1.07
**F4**	0.53	0.20	0.23	0.26
**5-FU**	0.14	0.14	12	0.02

**Table 8:** EC_50_ values of bacterial growth inhibition of **OE** and **F1**–**F4** obtained from *E. cavolini*.

Sample	EC_50_ (µg/mL)		
	*Escherichia coli*	*Pseudomonas aeruginosa*	*Staphylococcus aureus*
**OE**	>1000	>1000	514 ± 114
**F1**	422 ± 87	523 ± 42	417 ± 46
**F2**	255 ± 176	>1000	175 ± 25
**F3**	389 ± 135	>1000	171 ± 60
**F4**	797 ± 176	>1000	>1000
**Chloramphenicol**	0.75 ± 0.09	- *	1.50 ± 0.10

Values are expressed as mean value ± standard deviation of triplicate measurement. * Chloramphenicol is not effective against *P. aeruginosa*.

## Data Availability

The data are available from the corresponding authors upon reasonable request.

## Supplementary Materials

The HRMS and MS/MS were performed with different samples which spectra are available online. Figures S1–S5: HRMS spectra of **OE**, **F1**–**F4**, respectively. Figures S6–S20: MS/MS match between GNPS database (green) and *Eunicella cavolini* sample (black) for compounds 1–14 from Table 3, respectively.

## Sample Availability

Samples are available from the corresponding authors for a limited period.

## References

[1] Cochrane S.K.J., Andersen J.H., Berg T., Blanchet H., Borja A., Carstensen J., Elliott M., Hummel H., Niquil N., Renaud P.E. (2016). What is marine biodiversity? Towards common concepts and their implications for assessing biodiversity status. Front. Mar. Sci..

[2] Heiskanen A.S., Berg T., Uusitalo L., Teixeira H., Bruhn A., Krause-Jensen D., Lynam C.P., Rossberg A.G., Korpinen S., Uyarra M.C. (2016). Biodiversity in marine ecosystems-European developments toward robust assessments. Front. Mar. Sci..

[3] Bangmei X., Abbott I.A. (1987). Edible seaweeds of China and their place in the Chinese diet. Econ. Bot..

[4] Hamed I., Özogul F., Özogul Y., Regenstein J.M. (2015). Marine Bioactive Compounds and Their Health Benefits: A Review. Compr. Rev. Food Sci. Food Saf..

[5] Rocha J., Peixe L., Gomes N.C.M., Calado R. (2011). Cnidarians as a source of new marine bioactive compounds—An overview of the last decade and future steps for bioprospecting. Mar. Drugs.

[6] Ercolano G., De Cicco P., Ianaro A. (2019). New Drugs from the Sea: Pro-Apoptotic Activity of Sponges and Algae Derived Compounds. Mar. Drugs.

[7] Williams D.E., Andersen R.J. (2020). Biologically active marine natural products and their molecular targets discovered using a chemical genetics approach. Nat. Prod. Rep..

[8] Matulja D., Wittine K., Malatesti N., Laclef S., Turks M., Markovic M.K., Ambrožić G., Marković D. (2020). Marine natural products with high anticancer activities. Curr. Med. Chem..

[9] Park E.J., Pezzuto J.M. (2013). Antioxidant marine products in cancer chemoprevention. Antioxidants Redox Signal..

[10] Balakrishnan D., Kandasamy D., Nithyanand P. (2014). A review on antioxidant activity of marine organisms. Int. J. ChemTech Res..

[11] Florean C., Dicato M., Diederich M. (2020). Immune-modulating and anti-inflammatory marine compounds against cancer. Semin. Cancer Biol..

[12] Cheung R.C.F., Ng T.B., Wong J.H., Chen Y., Chan W.Y. (2016). Marine natural products with anti-inflammatory activity. Appl. Microbiol. Biotechnol..

[13] Choudhary A., Naughton L.M., Montánchez I., Dobson A.D.W., Rai D.K. (2017). Current status and future prospects of Marine Natural Products (MNPs) as antimicrobials. Mar. Drugs.

[14] Mayer A.M.S., Rodríguez A.D., Taglialatela-Scafati O., Fusetani N. (2017). Marine pharmacology in 2012–2013: Marine compounds with antibacterial, antidiabetic, antifungal, anti-inflammatory, antiprotozoal, antituberculosis, and antiviral activities; affecting the immune and nervous systems, and other miscellaneous mechanisms of. Mar. Drugs.

[15] Wittine K., Saftić L., Peršurić Ž., Pavelić S.K. (2019). Novel antiretroviral structures from marine organisms. Molecules.

[16] Petersen L.-E., Kellermann M.Y., Schupp P.J., Jungblut S., Liebich V., Bode-Dalby M. (2020). Secondary Metabolites of Marine Microbes: From Natural Products Chemistry to Chemical Ecology. YOUMARES 9-The Oceans: Our Research, Our Future.

[17] Carroll A.R., Copp B.R., Davis R.A., Keyzers R.A., Prinsep M.R. (2019). Marine natural products. Nat. Prod. Rep..

[18] Leal M.C., Calado R., Sheridan C., Alimonti A., Osinga R. (2013). Coral aquaculture to support drug discovery. Trends Biotechnol..

[19] Qi S.-H. (2012). Bioactive Compounds from Marine Gorgonian Corals. Stud. Nat. Prod. Chem..

[20] Changyun W., Haiyan L., Changlun S., Yanan W., Liang L., Huashi G. (2008). Chemical defensive substances of soft corals and gorgonians. Acta Ecol. Sin..

[21] Angiolillo M., Canese S., Duque C. (2018). Deep Gorgonians and Corals of the Mediterranean Sea. Corals in a Changing World.

[22] Weinbauer M.G., Velimirov B. (1998). Comparative morphometry of fan-like colonies of three Mediterranean gorgonians (Cnidaria: Gorgonacea). Cah. Biol. Mar..

[23] Weinbauer M.G., Velimirov B. (1995). Biomass and secondary production of the temperate gorgonian coral *Eunicella cavolini* (Coelenterata: Octocorallia). Mar. Ecol. Prog. Ser..

[24] Sánchez J.A., Dueñas L.F., Rowley S.J., Gonzalez-Zapata F.L., Vergara D.C., Montaño-Salazar S.M., Calixto-Botía I., Gómez C.E., Abeytia R., Colin P.L., Loya Y., Puglise K.A., Bridge T.C.L. (2019). Gorgonian Corals. Mesophotic Coral Ecosystems.

[25] Turicchia E., Abbiati M., Sweet M., Ponti M. (2018). Mass mortality hits gorgonian forests at Montecristo Island. Dis. Aquat. Organ..

[26] Rubio A.D.L.L., De Figueroa J.M.T., Rodríguez M.J.L., Tocino L.S. (2018). Mass mortality of *Eunicella singularis* (Anthozoa: Octocorallia) in the Chafarinas Islands (north Africa, western Mediterranean Sea). Rev. Biol. Mar. Oceanogr..

[27] Carella F., Aceto S., Saggiomo M., Mangoni O., De Vico G. (2014). Gorgonian disease outbreak in the Gulf of Naples: Pathology reveals cyanobacterial infection linked to elevated sea temperatures. Dis. Aquat. Organ..

[28] Sini M., Kipson S., Linares C., Koutsoubas D., Garrabou J. (2015). The yellow gorgonian *Eunicella cavolini*: Demography and disturbance levels across the Mediterranean Sea. PLoS ONE.

[29] Matulja D., Markovic M.K., Ambrožić G., Laclef S., Pavelić S.K., Marković D. (2020). Secondary metabolites from gorgonian corals of the genus *Eunicella*: Structural characterizations, biological activities, and synthetic approaches. Molecules.

[30] Khalifa S.A.M., Elias N., Farag M.A., Chen L., Saeed A., Hegazy M.E.F., Moustafa M.S., El-Wahed A.A., Al-Mousawi S.M., Musharraf S.G. (2019). Marine natural products: A source of novel anticancer drugs. Mar. Drugs.

[31] BioProCro–Center of excellence for Marine Bioprospecting. http://bioprocro.zci.hr/.

[32] WoRMS–World Register of Marine Species. http://www.marinespecies.org/aphia.php?p=stats.

[33] Ioannou E., Abdel-Razik A.F., Zervou M., Christofidis D., Alexi X., Vagias C., Alexis M.N., Roussis V. (2009). 5alpha,8alpha-Epidioxysterols from the gorgonian *Eunicella cavolini* and the ascidian *Trididemnum inarmatum*: Isolation and evaluation of their antiproliferative activity. Steroids.

[34] Ioannou E., Abdel-Razik A.F., Alexi X., Vagias C., Alexis M.N., Roussis V. (2009). 9,11-Secosterols with antiproliferative activity from the gorgonian *Eunicella cavolini*. Bioorganic Med. Chem..

[35] Ioannou E., Abdel-Razik A.F., Alexi X., Vagias C., Alexis M.N., Roussis V. (2008). Pregnanes with antiproliferative activity from the gorgonian *Eunicella cavolini*. Tetrahedron.

[36] Hall-Spencer J.M., Pike J., Munn C.B. (2007). Diseases affect cold-water corals too: *Eunicella verrucosa* (Cnidaria: Gorgonacea) necrosis in SW England. Dis. Aquat. Organ..

[37] Heikoop J.M., Hickmott D.D., Risk M.J., Shearer C.K., Atudorei V. (2002). Potential climate signals from the deep-sea gorgonian coral *Primnoa resedaeformis*. Hydrobiologia.

[38] Cimino G., De Rosa S., De Stefano S. (1984). Antiviral agents from a gorgonian, *Eunicella cavolini*. Experientia.

[39] Cimino G., Desiderio B., De Stefano S., Sodano G. (1979). Chemistry of mediterranean gorgonians. II. Pregna 4,20-dien-11α-ol-3-one acetate, a novel steroid from the gorgonian Eunicella cavolini. Experientia.

[40] Mancini I., Guella G., Zibrowius H., Laurent D., Pietra F. (1999). A novel type of a second epoxy bridge in eunicellane diterpenes: Isolation and characterization of massileunicellins A-C from the gorgonian *Eunicella cavolinii*. Helv. Chim. Acta.

[41] Mancini I., Guella G., Zibrowius H., Pietra F. (2000). Configuration, conformation, and reactivity of highly functionalized eunicellane diterpenes isolated from the gorgonians *Eunicella cavolinii* and *Eunicella singularis* from Marseille. Helv. Chim. Acta.

[42] Edrada R.A., Wray V., Handayani D., Schupp P., Balbin-Oliveros M., Proksch P. (2000). Structure-activity relationships of bioactive metabolites from some Indo-Pacific marine invertebrates. Stud. Nat. Prod. Chem..

[43] Bracco S., Fumagalli P., Fusi P., Santambrogio C., Rolandi V., Brajkovic A. (2016). Identification of the chromophores in *Corallium rubrum* gem quality corals by HPLC/UV, ESI-MS and 1H NMR spectroscopy. Period. di Mineral..

[44] Weng A., Thakur M., Melzig F.M., Fuchs H. (2011). Chemistry and pharmacology of saponins: Special focus on cytotoxic properties. Bot. Targets Ther..

[45] Khan H., Saeedi M., Nabavi S.M., Mubarak M.S., Bishayee A. (2018). Glycosides from Medicinal Plants as Potential Anticancer Agents: Emerging Trends Towards Future Drugs. Curr. Med. Chem..

[46] Thomas N.V., Kim S.K. (2011). Potential pharmacological applications of polyphenolic derivatives from marine brown algae. Environ. Toxicol. Pharmacol..

[47] Deghrigue M., Dellai A., Akremi N., Le Morvan V., Robert J., Bouraoui A. (2013). Evaluation of antiproliferative and antioxidant activities of the organic extract and its polar fractions from the Mediterranean gorgonian *Eunicella singularis*. Environ. Toxicol. Pharmacol..

[48] Prior R.L., Wu X., Schaich K. (2005). Standardized methods for the determination of antioxidant capacity and phenolics in foods and dietary supplements. J. Agric. Food Chem..

[49] Cvejic J., Tambutté S., Lotto S., Mikov M., Slacanin I., Allemand D. (2007). Determination of canthaxanthin in the red coral (*Corallium rubrum*) from Marseille by HPLC combined with UV and MS detection. Mar. Biol..

[50] Shahbudin S., Deny S., Zakirun A.M.T., Haziyamm T.A.H., Akbar John B., Taher M. (2011). Antioxidant properties of soft coral *Dendronephthya* sp. Int. J. Pharmacol..

[51] Mydlarz L.D., Harvell C.D. (2007). Peroxidase activity and inducibility in the sea fan coral exposed to a fungal pathogen. Comp. Biochem. Physiol. A Mol. Integr. Physiol..

[52] Yost D.M., Jones R.J., Mitchelmore C.L. (2010). Alterations in dimethylsulfoniopropionate (DMSP) levels in the coral *Montastraea franksi* in response to copper exposure. Aquat. Toxicol..

[53] Toledo-Hernández C., Ruiz-Diaz C.P., Díaz-Vázquez L.M., Santiago-Cárdenas V., Rosario-Berrios D.N., García-Almedina D.M., Roberson L.M., Ambiente S., Sam M., Juan S. (2017). Comparison of chemical compounds associated with sclerites from healthy and diseased sea fan corals (*Gorgonia ventalina*). PeerJ.

[54] Downs C.A., Fauth J.E., Halas J.C., Dustan P., Bemiss J., Woodley C.M. (2002). Oxidative stress and seasonal coral bleaching. Free Radic. Biol. Med..

[55] Griffin S.P., Bhagooli R. (2004). Measuring antioxidant potential in corals using the FRAP assay. J. Exp. Mar. Biol. Ecol..

[56] Sang V.T., Dat T.T.H., Vinh L.B., Cuong L.C.V., Oanh P.T.T., Ha H., Kim Y.H., Anh H.L.T., Yang S.Y. (2019). Coral and coral-associated microorganisms: A prolific source of potential bioactive natural products. Mar. Drugs.

[57] Deghrigue M., Dellai A., Bouraoui A., Akremi N., Le Morvan V., Robert J., Bouraoui A. (2013). In Vitro Antiproliferative and Antioxidant Activities of the Organic Extract and Its Semi-Purified Fractions from the Mediterranean Gorgonian *Eunicella Singularis*. Int. J. Pharm. Pharm. Sci..

[58] Young A.J., Lowe G.L. (2018). Carotenoids—antioxidant properties. Antioxidants.

[59] Gonzalez-Burgos E., Gomez-Serranillos M.P. (2012). Terpene Compounds in Nature: A Review of Their Potential Antioxidant Activity. Curr. Med. Chem..

[60] Apak R., Gorinstein S., Böhm V., Schaich K.M., Özyürek M., Güçlü K. (2013). Methods of measurement and evaluation of natural antioxidant capacity/activity (IUPAC technical report). Pure Appl. Chem..

[61] San Miguel-Chávez R., Soto-Hernández M., Palma-Tenango M., García-Mateos R. (2017). Phenolic Antioxidant Capacity: A Review of the State of the Art. Phenolic Compounds—Biological Activity.

[62] Huang D., Boxin O.U., Prior R.L. (2005). The chemistry behind antioxidant capacity assays. J. Agric. Food Chem..

[63] Deghrigue M., Festa C., Ghribi L., D’Auria M.V., De Marino S., Ben Jannet H., Bouraoui A. (2015). Anti-inflammatory and analgesic activities with gastroprotective effect of semi-purified fractions and isolation of pure compounds from Mediterranean gorgonian *Eunicella singularis*. Asian Pac. J. Trop. Med..

[64] Deghrigue M., Festa C., Ghribi L., D’auria M.V., de Marino S., Ben Jannet H., Ben Said R., Bouraoui A. (2014). Pharmacological evaluation of the semi-purified fractions from the soft coral *Eunicella singularis* and isolation of pure compounds. Daru J. Pharm. Sci..

[65] Bruno F., Spaziano G., Liparulo A., Roviezzo F., Nabavi S.M., Sureda A., Filosa R., D’Agostino B. (2018). Recent advances in the search for novel 5-lipoxygenase inhibitors for the treatment of asthma. Eur. J. Med. Chem..

[66] Osman N.I., Sidik N.J., Awal A., Adam N.A.M., Rezali N.I. (2016). In vitro xanthine oxidase and albumin denaturation inhibition assay of *Barringtonia racemosa* L. And total phenolic content analysis for potential anti-inflammatory use in gouty arthritis. J. Intercult. Ethnopharmacol..

[67] Williams L.A.D., O’Connar A., Latore L., Dennis O., Ringer S., Whittaker J.A., Conrad J., Vogler B., Rosner H., Kraus W. (2008). The in vitro anti-denaturation effects induced by natural products and non-steroidal compounds in heat treated (Immunogenic) bovine serum albumin is proposed as a screening assay for the detection of anti-inflammatory compounds, without the use of animals. West. Indian Med. J..

[68] Muthiyan R., Mahanta N., Nambikkairaj B., Immanuel T., De A. (2018). Antioxidant and anti-inflammatory effects of a methanol extract from the marine sponge *Hyrtios erectus*. Pharmacogn. Mag..

[69] Bouhlali E.D.T., El Hilaly J., Ennassir J., Benlyas M., Alem C., Amarouch M.Y., Filali-Zegzouti Y. (2018). Anti-inflammatory properties and phenolic profile of six Moroccan date fruit (*Phoenix dactylifera* L.) varieties. J. King Saud Univ.Sci..

[70] Sircar J.C., Schwender C.F., Johnson E.A. (1983). Soybean lipoxygenase inhibition by nonsteroidal antiinflammatory drugs. Prostaglandins.

[71] Wei W.C., Sung P.J., Duh C.Y., Chen B.W., Sheu J.H., Yang N.S. (2013). Anti-inflammatory activities of natural products isolated from soft corals of Taiwan between 2008 and 2012. Mar. Drugs.

[72] Lei H. (2016). Diterpenoids of Gorgonian Corals: Chemistry and Bioactivity. Chem. Biodivers..

[73] González Y., Torres-Mendoza D., Jones G.E., Fernandez P.L. (2015). Marine Diterpenoids as Potential Anti-Inflammatory Agents. Mediat. Inflamm..

[74] Souza C.R.M., Bezerra W.P., Souto J.T. (2020). Marine Alkaloids with Anti-Inflammatory Activity: Current Knowledge and Future Perspectives. Mar. Drugs.

[75] Jacquot C., McGinley C.M., Plata E., Holman T.R., Van Der Donk W.A. (2008). Synthesis of 11-thialinoleic acid and 14-thialinoleic acid, inhibitors of soybean and human lipoxygenases. Org. Biomol. Chem..

[76] Wyld L., Smith O., Lawry J., Reed M.W.R., Brown N.J. (1998). Cell cycle phase influences tumour cell sensitivity to aminolaevulinic acid-induced photodynamic therapy in vitro. Br. J. Cancer.

[77] Badisa R.B., Darling-Reed S.F., Joseph P., Cooperwood J.S., Latinwo L.M., Goodman C.B. (2009). Selective Cytotoxic Activities of Two Novel Synthetic Drugs on Human Breast Carcinoma MCF-7 Cells. Anticancer Res..

[78] Rashidi M., Seghatoleslam A., Namavari M., Amiri A., Fahmidehkar M.A., Ramezani A., Eftekhar E., Hosseini A., Erfani N., Fakher S. (2017). Selective cytotoxicity and apoptosis-induction of *Cyrtopodion scabrum* extract against digestive cancer cell lines. Int. J. Cancer Manag..

[79] Satomi Y. (2017). Antitumor and Cancer-preventative Function of Fucoxanthin: A Marine Carotenoid. Anticancer Res..

[80] Kim K. (1994). Antimicrobial activity in gorgonian corals (Coelenterata, Octocorallia). Coral Reefs.

[81] Kim K., Kim P.D., Alker A.P., Harvell C.D. (2000). Chemical resistance of gorgonian corals against fungal infections. Mar. Biol..

[82] Kim K., Harvell C.D., Kim P.D., Smith G.W., Merkel S.M. (2000). Fungal disease resistance of caribbean sea fan corals (*Gorgonia* spp.). Mar. Biol..

[83] Jensen P.R., Harvell C.D., Wirtz K., Fenical W. (1996). Antimicrobial activity of extracts of Caribbean gorgonian corals. Mar. Biol..

[84] Alker A.P., Smith G.W., Kim K. (2001). Characterization of *Aspergillus sydowii* (Thom et Church), a fungal pathogen of Caribbean sea fan corals. Hydrobiologia.

[85] Alker A.P., Kim K., Dube D.H., Harvell C.D. (2004). Localized induction of a generalized response against multiple biotic agents in Caribbean sea fans. Coral Reefs.

[86] Liang L.F., Wang X.J., Zhang H.Y., Liu H.L., Li J., Lan L.F., Zhang W., Guo Y.W. (2013). Bioactive polyhydroxylated steroids from the Hainan soft coral *Sinularia depressa* Tixier-Durivault. Bioorganic Med. Chem. Lett..

[87] Bayer T., Arif C., Ferrier-Pagès C., Zoccola D., Aranda M., Voolstra C.R. (2013). Bacteria of the genus *Endozoicomonas* dominate the microbiome of the Mediterranean gorgonian coral *Eunicella cavolini*. Mar. Ecol. Prog. Ser..

[88] Bally M., Garrabou J. (2007). Thermodependent bacterial pathogens and mass mortalities in temperate benthic communities: A new case of emerging disease linked to climate change. Glob. Chang. Biol..

[89] Baier R.E., Meyer A.E., Lee L.H. (1991). Aspects of Bioadhesion. Fundamentals of Adhesion.

[90] Hunt L.R., Smith S.M., Downum K.R., Mydlarz L.D. (2012). Microbial regulation in gorgonian corals. Mar. Drugs.

[91] Shannon E., Abu-Ghannam N. (2016). Antibacterial derivatives of marine algae: An overview of pharmacological mechanisms and applications. Mar. Drugs.

[92] Liu Z., Sun X., Sun X., Wang S., Xu Y. (2019). Fucoxanthin Isolated from *Undaria pinnatifida* Can Interact with *Escherichia coli* and *lactobacilli* in the Intestine and Inhibit the Growth of Pathogenic Bacteria. J. Ocean. Univ. China.

[93] Samorì C., Costantini F., Galletti P., Tagliavini E., Abbiati M. (2018). Inter- and Intraspecific Variability of Nitrogenated Compounds in Gorgonian Corals via Application of a Fast One-Step Analytical Protocol. Chem. Biodivers..

[94] Kodzius R., Gojobori T. (2015). Marine metagenomics as a source for bioprospecting. Mar. Genom..

[95] Edeoga H.O., Okwu D.E., Mbaebie B.O. (2005). Phytochemical constituents of some Nigerian medicinal plants. Afr. J. Biotechnol..

[96] Taga M.S., Miller E.E., Pratt D.E. (1984). Chia seeds as a source of natural lipid antioxidants. J. Am. Oil Chem. Soc..

[97] Ghorai N., Chakraborty S., Gucchait S., Saha S.K., Biswas S. (2012). Estimation of total Terpenoids concentration in plant tissues using a monoterpene, Linalool as standard reagent. Protoc. Exch..

[98] Wang M., Carver J.J., Phelan V.V., Sanchez L.M., Garg N., Peng Y., Nguyen D.D., Watrous J., Kapono C.A., Luzzatto-Knaan T. (2017). Sharing and community curation of mass spectrometry data with GNPS. Nat. Biotechnol..

[99] Kim J.K., Noh J.H., Lee S., Choi J.S., Suh H., Chung H.Y., Song Y.O., Choi W.C. (2002). The first total synthesis of 2,3,6-tribromo-4,5-dihydroxybenzyl methyl ether (TDB) and its antioxidant activity. Bull. Korean Chem. Soc..

[100] Zheleva-Dimitrova D., Nedialkov P., Kitanov G. (2010). Radical scavenging and antioxidant activities of methanolic extracts from *Hypericum* species growing in Bulgaria. Pharmacogn. Mag..

[101] Oso B., Karigidi K. (2019). Inhibitory action of dried leaf of *Cassia alata* (Linn.) Roxb against lipoxygenase activity and nitric oxide generation. Sci. Agropecu..

[102] Gazivoda T., Raić-Malić S., Krištafor V., Makuc D., Plavec J., Bratulić S., Kraljević-Pavelić S., Pavelić K., Naesens L., Andrei G. (2008). Synthesis, cytostatic and anti-HIV evaluations of the new unsaturated acyclic C-5 pyrimidine nucleoside analogues. Bioorganic Med. Chem..

[103] CLSI (2015). M07-A10 Methods for Dilution Antimicrobial Susceptibility Tests for Bacteria That Grow Aerobically; Approved Standard-Tenth Edition.

[104] Ritz C., Baty F., Streibig J.C., Gerhard D. (2015). Dose-response analysis using R. PLoS ONE.

