# Supplementary material for: Chemical Evaluation, Antioxidant, Antiproliferative, Anti-Inflammatory and Antibacterial Activities of Organic Extract and Semi-Purified Fractions of the Adriatic Sea Fan, Eunicella cavolini
Source: Molecules. 2021 Sep 23;26(19):5751. doi: 10.3390/molecules26195751 (PMC8510138; doi:10.3390/molecules26195751)
Supplement: Supplementary file 1 [file molecules-26-05751-s001.zip › molecules-1386383-supplementary.pdf]

Supplementary material

Chemical Evaluation, Antioxidant, Antiproliferative, Anti-Inflammatory and Antibacterial Activities of Organic Extract and Semi-purified Fractions of the Adriatic Sea Fan, *Eunicella cavolini*

Dario Matulja[1], Petra Grbčić[1], Krunoslav Bojanić[2], Natalija Topić-Popović[2], Rozelindra Čož-Rakovac[2], Sylvain Laclef[3], Tomislav Šmuc[2], Ozren Jović[2], Dean Marković[1]*, Sandra Kraljević Pavelić[4]*

1 Department of Biotechnology, University of Rijeka, Radmile Matejčić 2, 51000 Rijeka, Croatia; dario.matulja@biotech.uniri.hr (Dario Matulja); petra.grbcic@biotech.uniri.hr (P.G.); dean.markovic@biotech.uniri.hr (D.M.)

2 Ruđer Bošković Institute, Bijenička cesta 54, 10000 Zagreb, Croatia; Krunoslav.Bojanic@irb.hr (K.B.); Natalija.Topic.Popovic@irb.hr (N.T.P.); Rozelindra.Coz-Rakovac@irb.hr (R.Č.-R.); tomislav.smuc@irb.hr (T.Š.), ozren.jovic@irb.hr (O.J.)

3 Laboratoire de Glycochimie, des Antimicrobiens et des Agroressources (LG2A) UMR CNRS 7378 - Ins-titut de Chimie de Picardie FR 3085, Université de Picardie Jules Verne, 33 rue Saint Leu, 80039 Amiens CEDEX, France; sylvain.laclef@u-picardie.fr (S.L.)

4 Faculty of Health Studies, University of Rijeka, Viktora Cara Emina 5, Rijeka, 51000, Croatia; sandrakp@uniri.hr

* Correspondence: sandrakp@uniri.hr (S.K.P.); dean.markovic@biotech.uniri.hr (D.M.); +385-51- 688 266 (S.K.P.); +385-91-500-8676 (D.M.)

The HRMS and MS/MS were performed with different samples which spectra are available online at www.mdpi.com/xxx/s1. Figures S1-S5: HRMS spectra of OE, F1-F4, respectively. Figures S6-S20: MS/MS match between GNPS database (green) and *Eunicella cavolini* sample (black) for compounds 1-15 from Table 3, respectively.

Mass Spectroscopy of the different fractions:

HRMS of the different fractions were performed using a SYNAPT G2-Si Q-TOF hybrid quadrupole time-of-flight instrument (Waters-Micromass, Manchester, UK), equipped with an electrospray (ESI) ionisation source (Z-spray) and an additional sprayer for the reference compound (Lock Spray). The samples were prepared by dissolving 1 mg of the different fraction in MeOH. The sample (1 µL of injection volume) were loaded on a KITETEX C_{18} (1.3 µm , 50 x 2.1 mm) column (Phenomenex, Torrance, CA, USA) heated at 50 °C. The effluent was fully directed toward the ESI source of the Q-TOF instrument. ESI-HRMS data were recorded in the positive ion modes. The source and desolvation temperatures were 120 and 450 °C, respectively. Nitrogen was used as a drying and nebulizing gas at flow rates of 50 and 900 L/h, respectively. Typically, the capillary voltage was 3 kV, the sampling cone voltage 20 V and the source offset 20 V. Lock mass corrections using $[M+H]^+$, $[M-H]^-$ ions at *m/z* 556.2771 and 554.2615 of a leucine-enkephalin solution (50 pg μL^{-1} in 50:50 acetonitrile/water + 0.1% formic acid) were applied for accurate mass measurements (elemental composition determination). The mass range was 50–1150 Da and spectra were recorded at 0.2 s/scan in the centroid mode at a resolution of 20,000 (FWHM) in the sensitivity mode. Data acquisition and processing were performed with MassLynx 4.1 software.

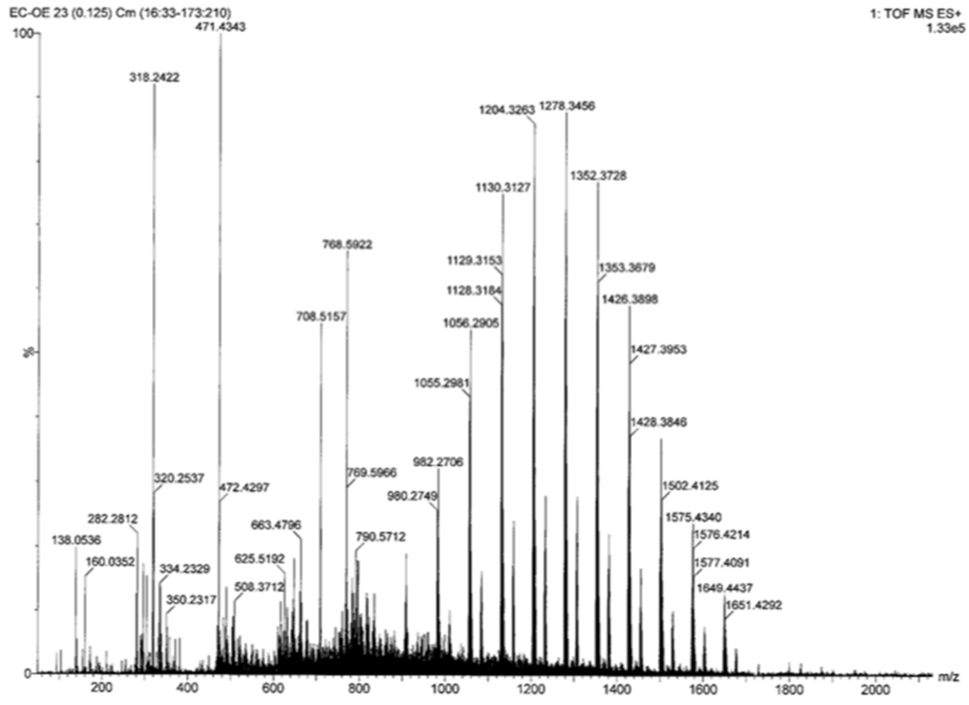

Figure S1. HRMS spectrum of OE.

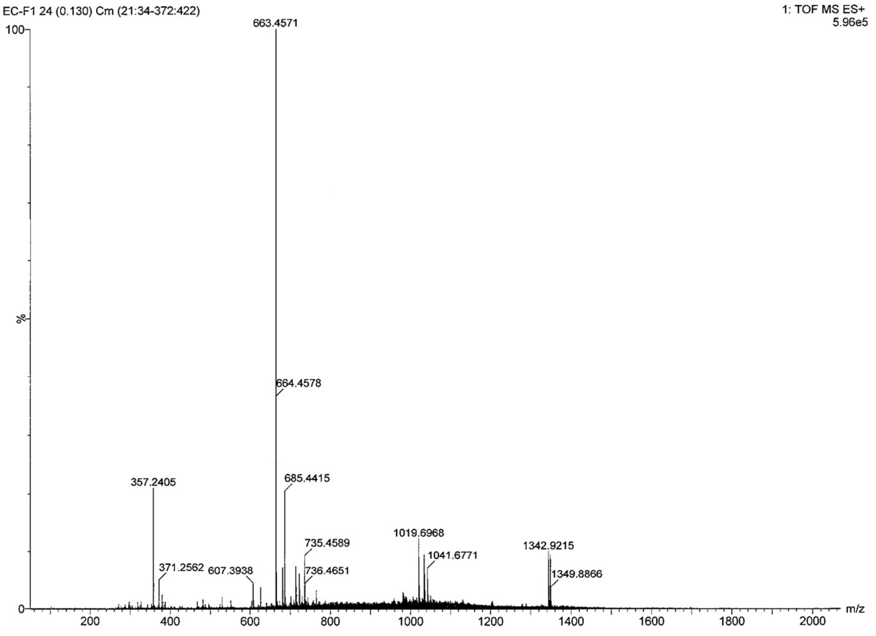

Figure S2. HRMS spectrum of **F1**.

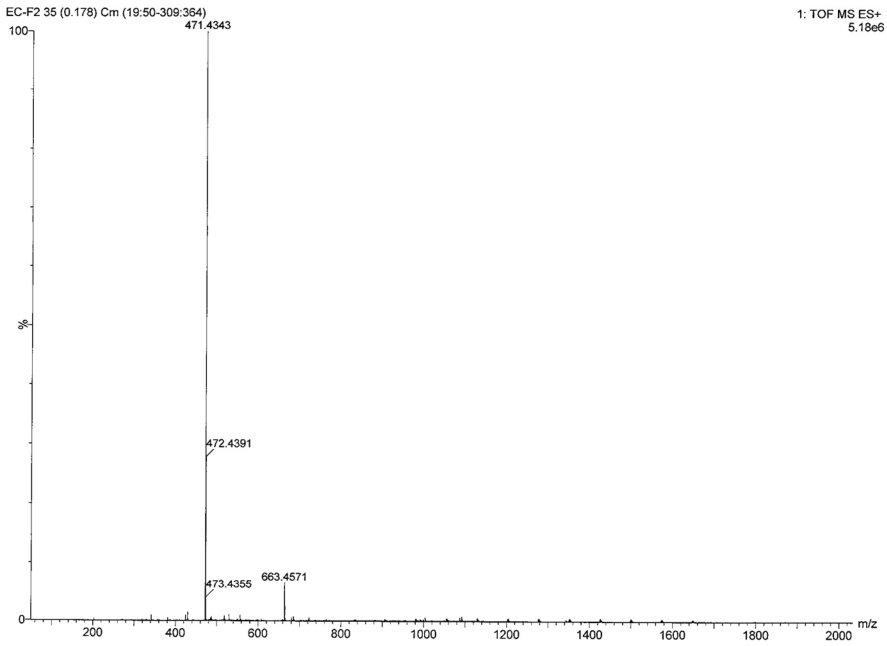

Figure S3. HRMS spectrum of **F2**.

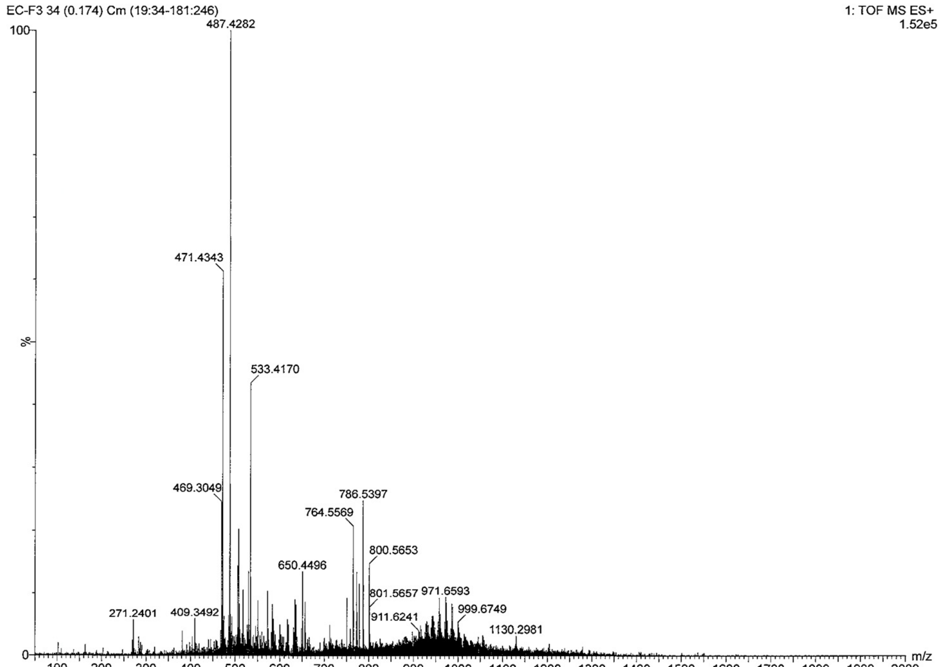

Figure S4. HRMS spectrum of **F3**.

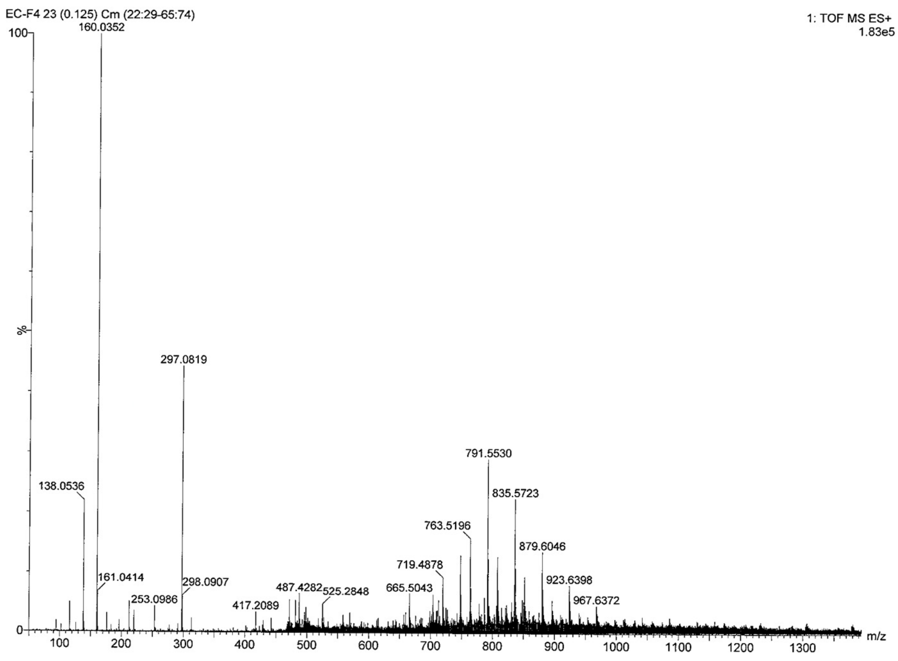

Figure S5. HRMS spectrum of **F4**.

GNPS dereplication

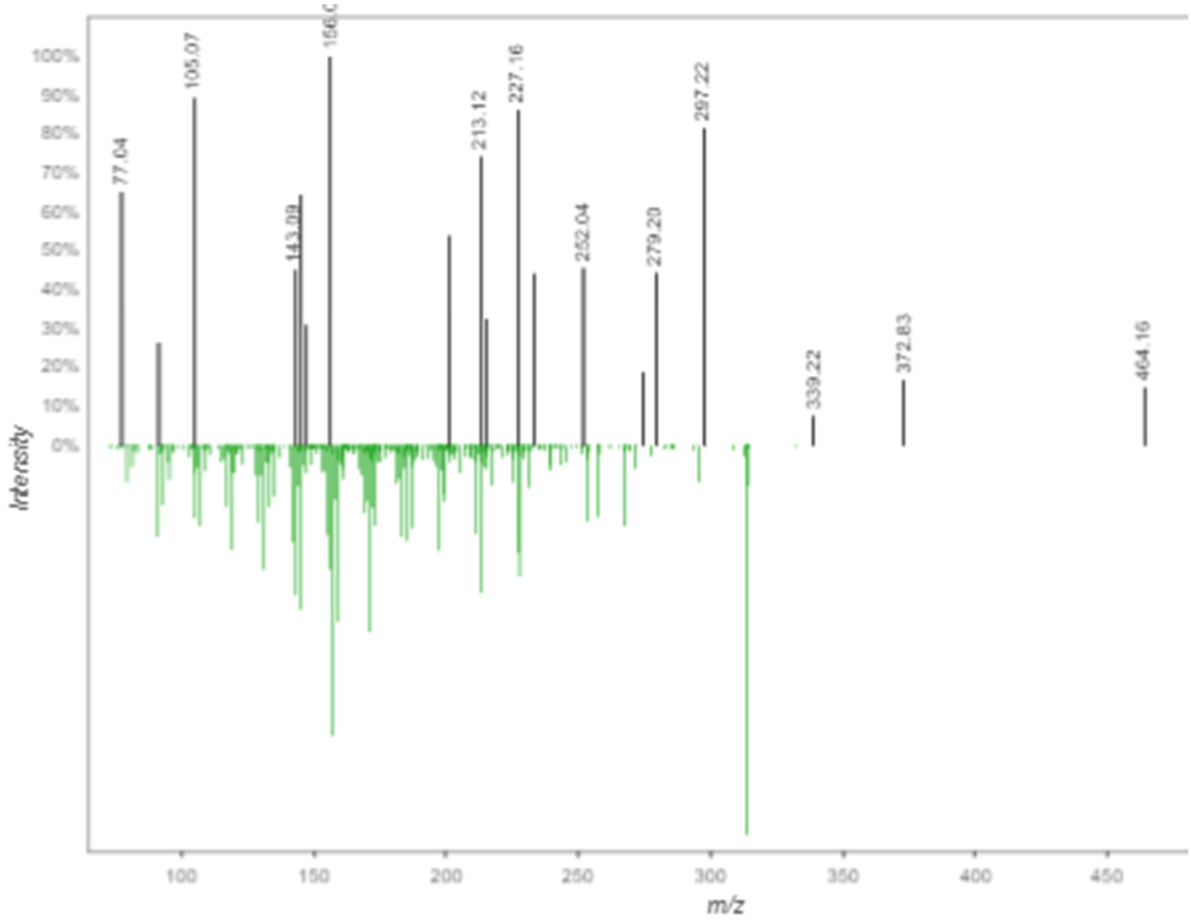

Figure S6: MS/MS match between GNPS database (green) and *Eunicella cavolini* sample (black) for compound 1.

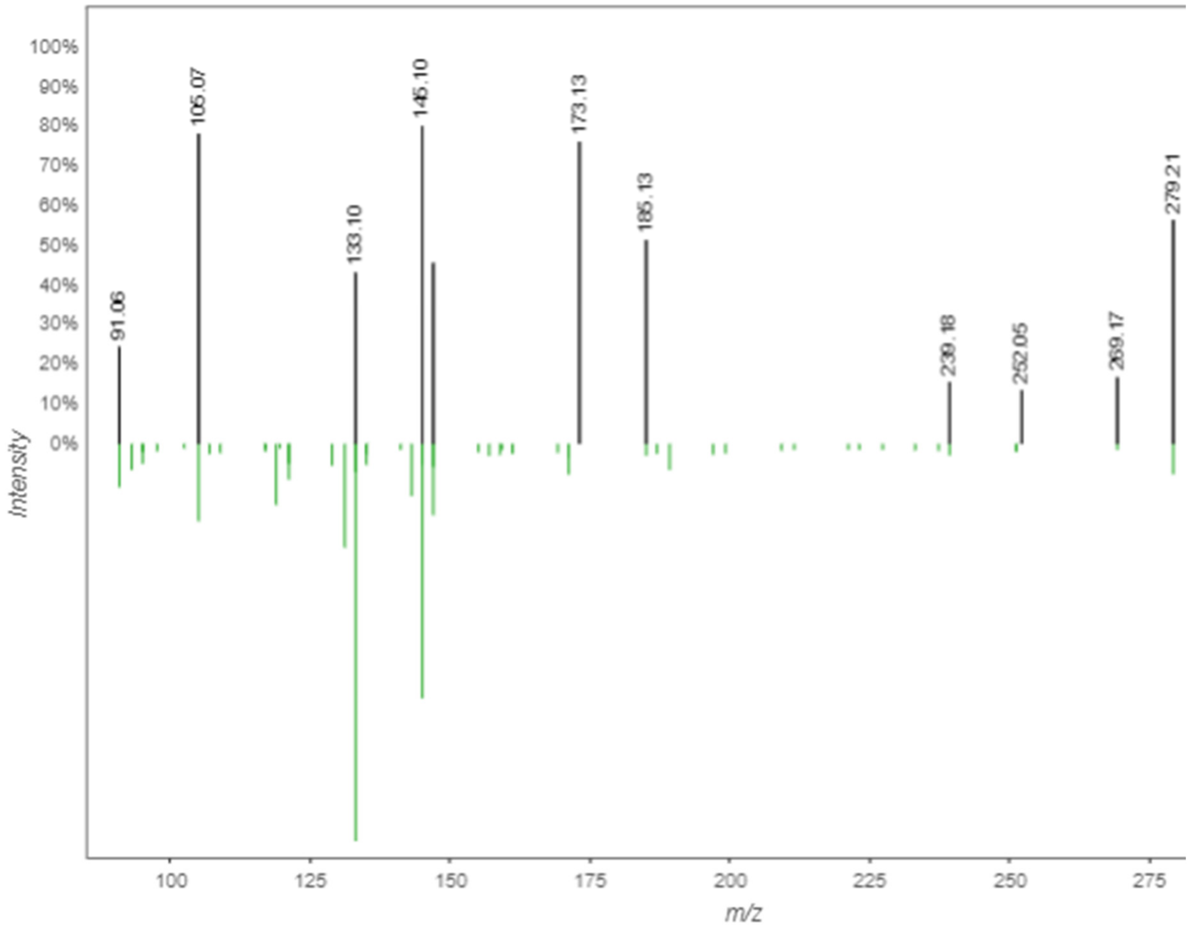

Figure S7: MS/MS match between GNPS database (green) and *Eunicella cavolini* sample (black) for compound 2.

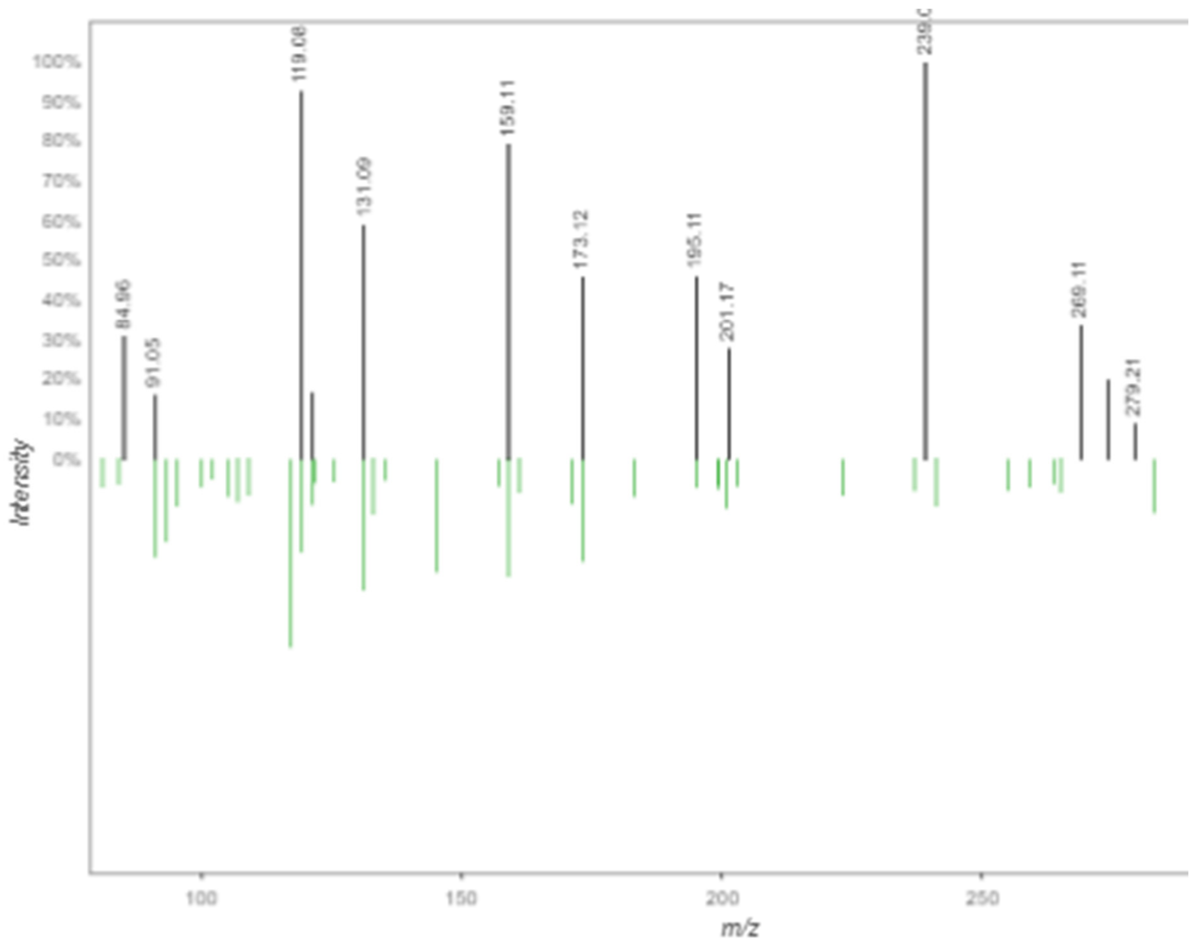

Figure S8: MS/MS match between GNPS database (green) and *Eunicella cavolini* sample (black) for compound 3.

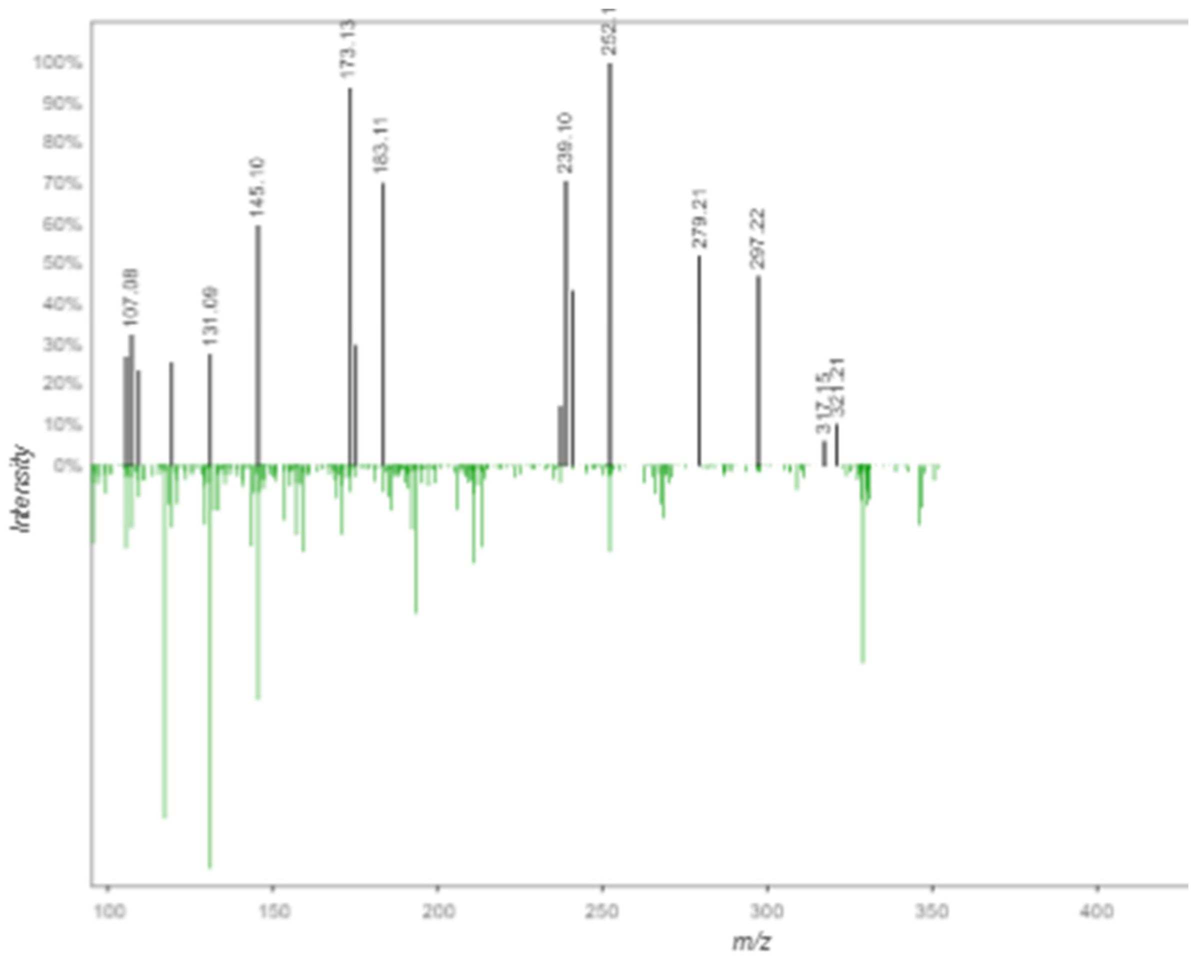

Figure S9: MS/MS match between GNPS database (green) and *Eunicella cavolini* sample (black) for compound 4.

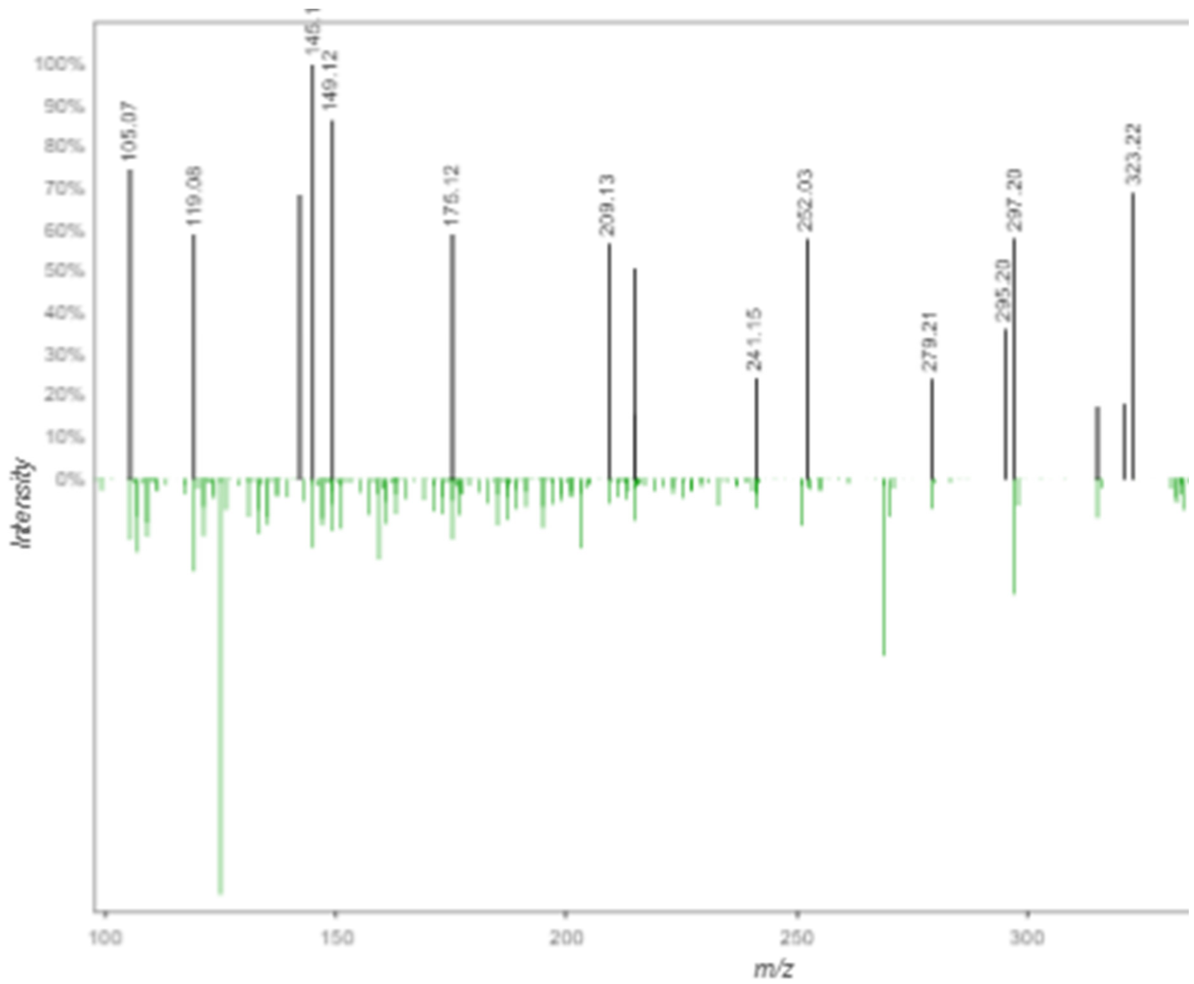

Figure S10: MS/MS match between GNPS database (green) and *Eunicella cavolini* sample (black) for compound 5.

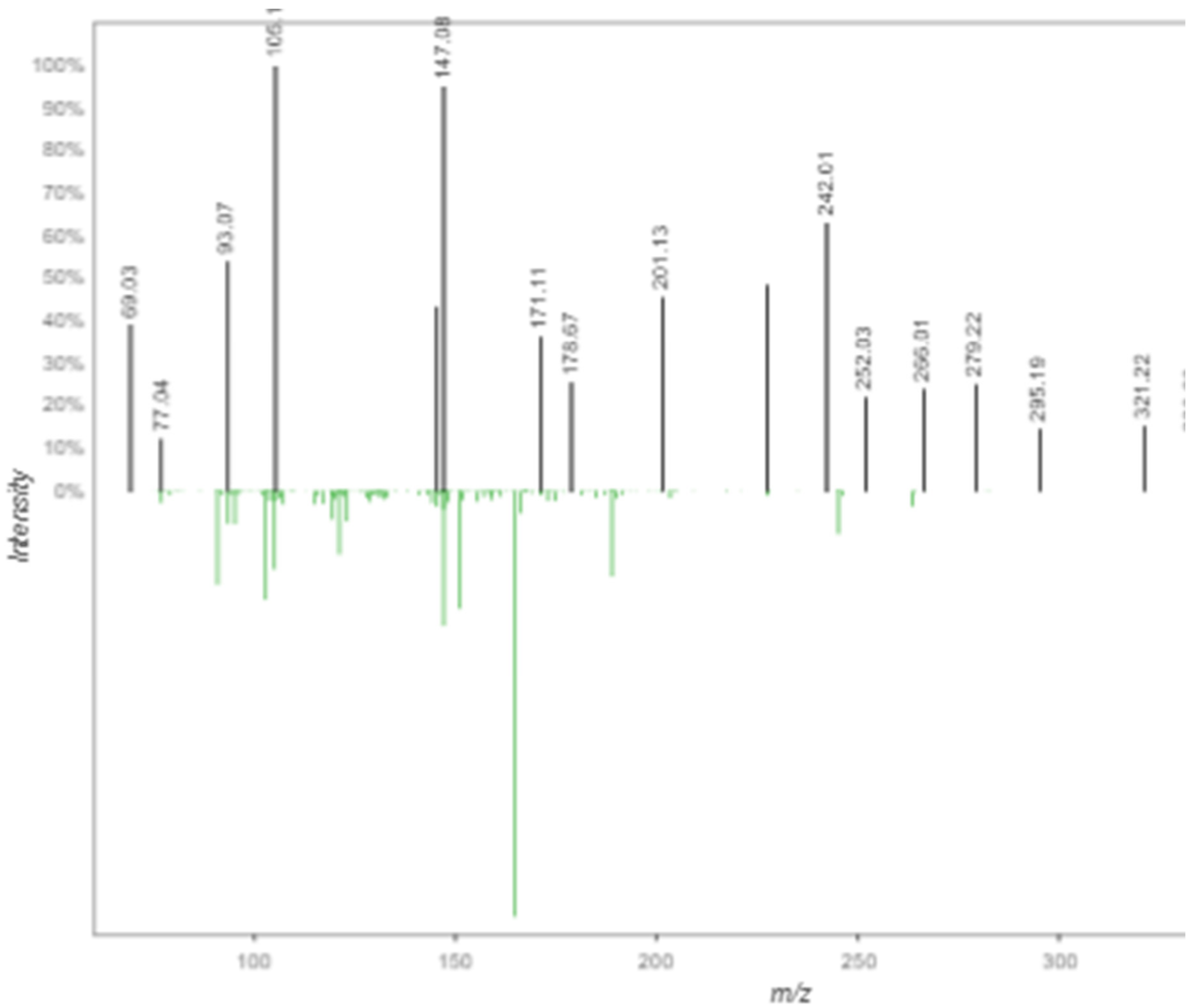

Figure S11: MS/MS match between GNPS database (green) and *Eunicella cavolini* sample (black) for compound 6.

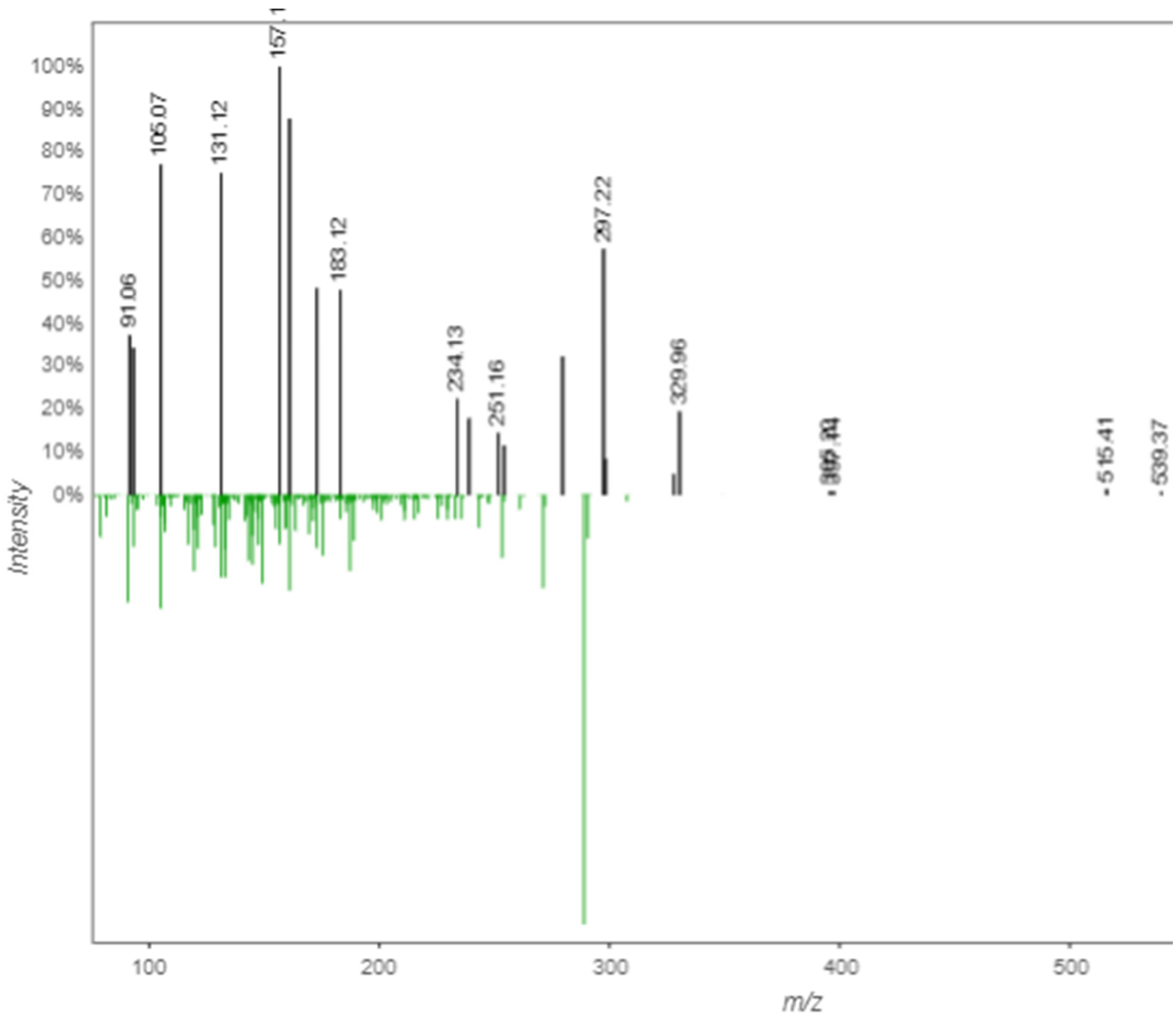

Figure S12: MS/MS match between GNPS database (green) and *Eunicella cavolini* sample (black) for compound 7.

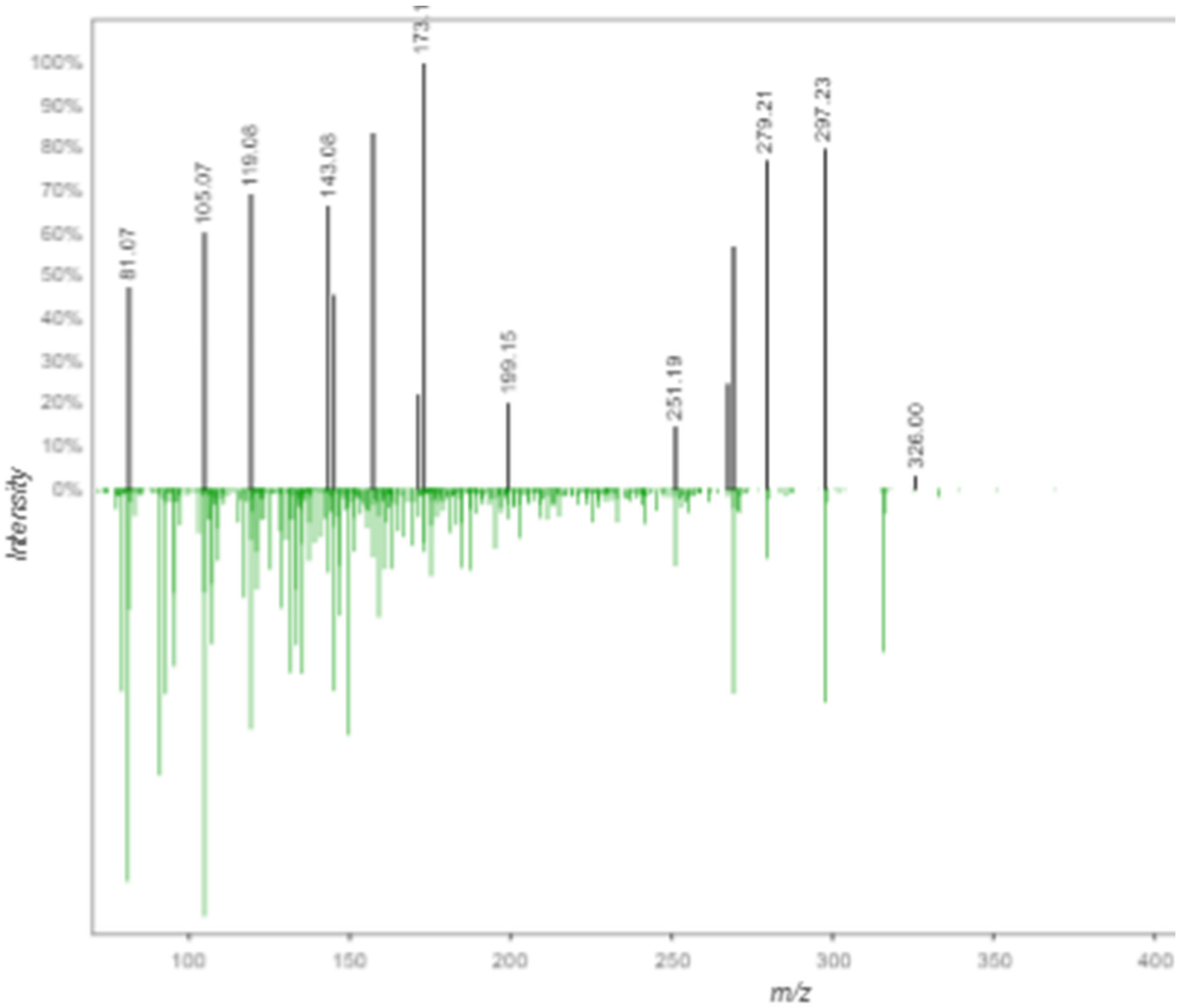

Figure S13: MS/MS match between GNPS database (green) and *Eunicella cavolini* sample (black) for compound 8.

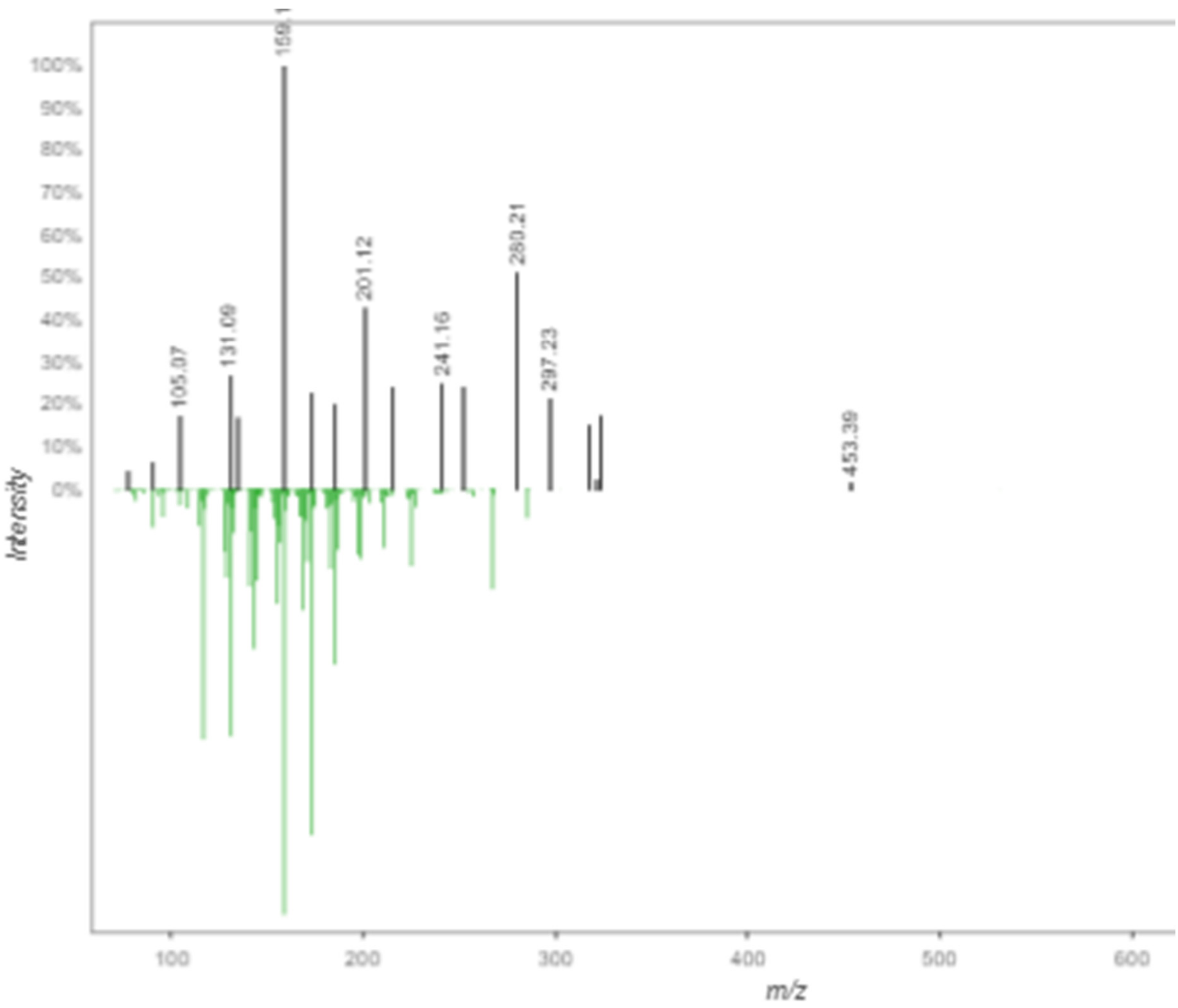

Figure S14: MS/MS match between GNPS database (green) and *Eunicella cavolini* sample (black) for compound 9.

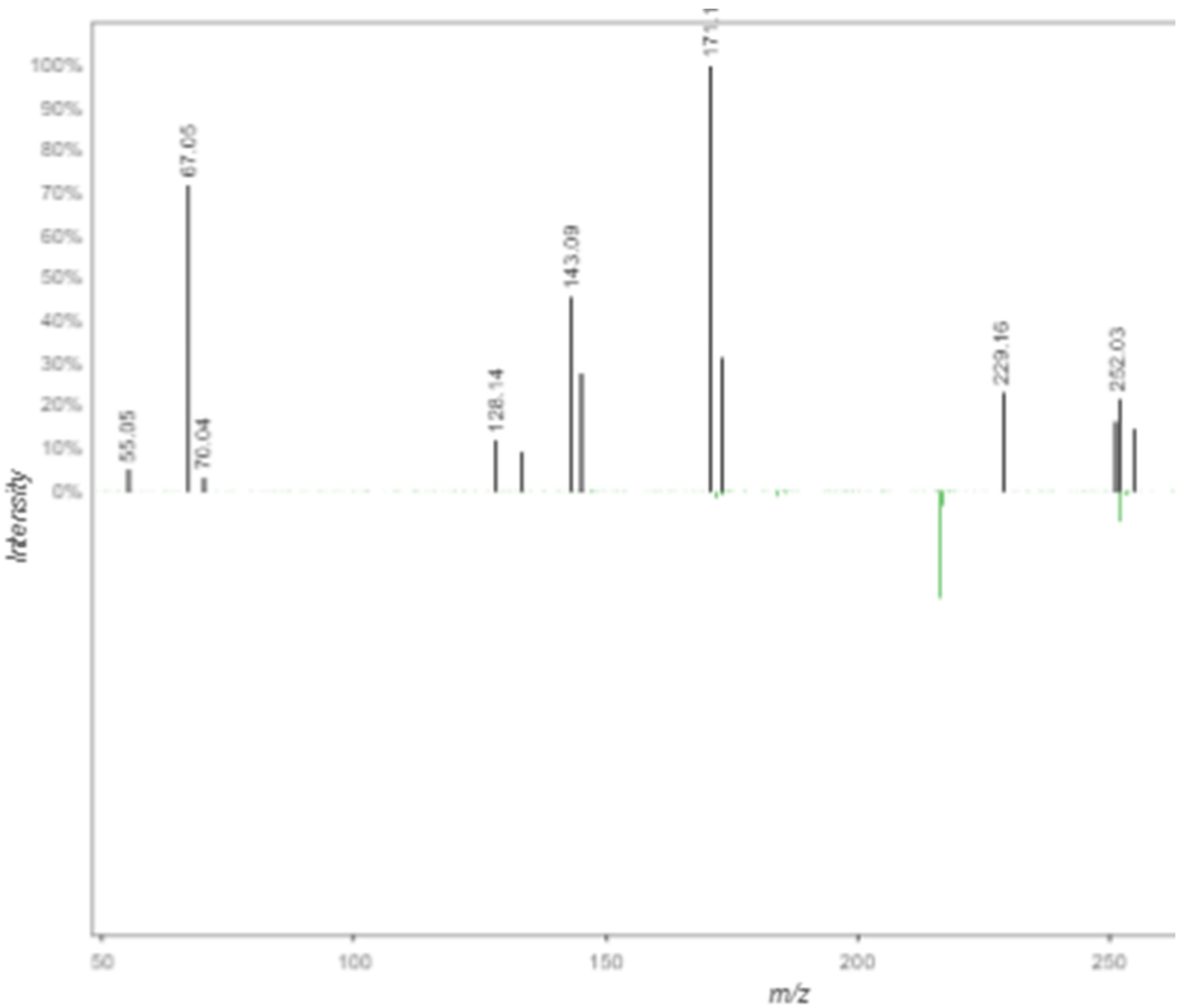

Figure S15: MS/MS match between GNPS database (green) and *Eunicella cavolini* sample (black) for compound 10.

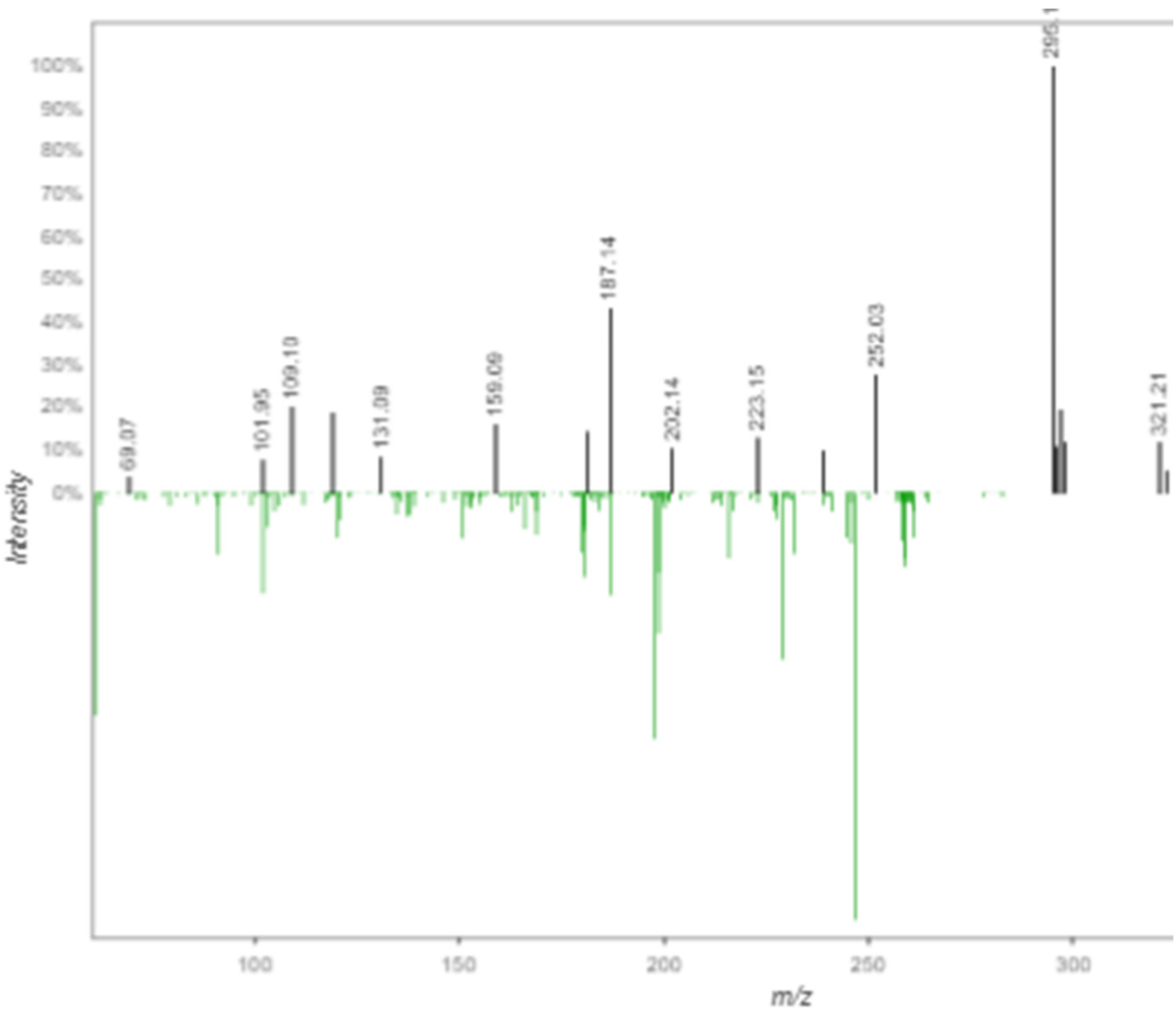

Figure S16: MS/MS match between GNPS database (green) and *Eunicella cavolini* sample (black) for compound 11.

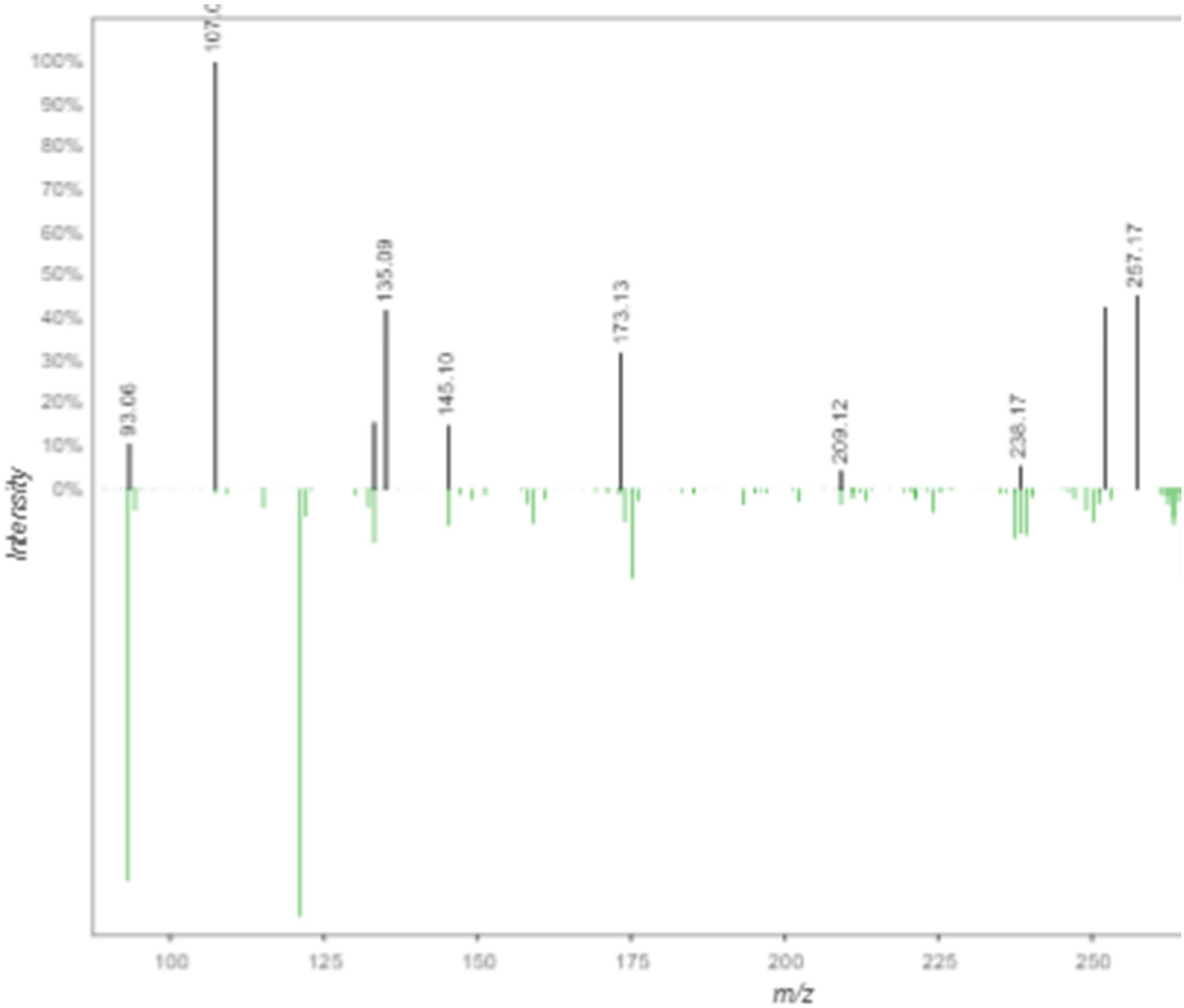

Figure S17: MS/MS match between GNPS database (green) and *Eunicella cavolini* sample (black) for compound 12.

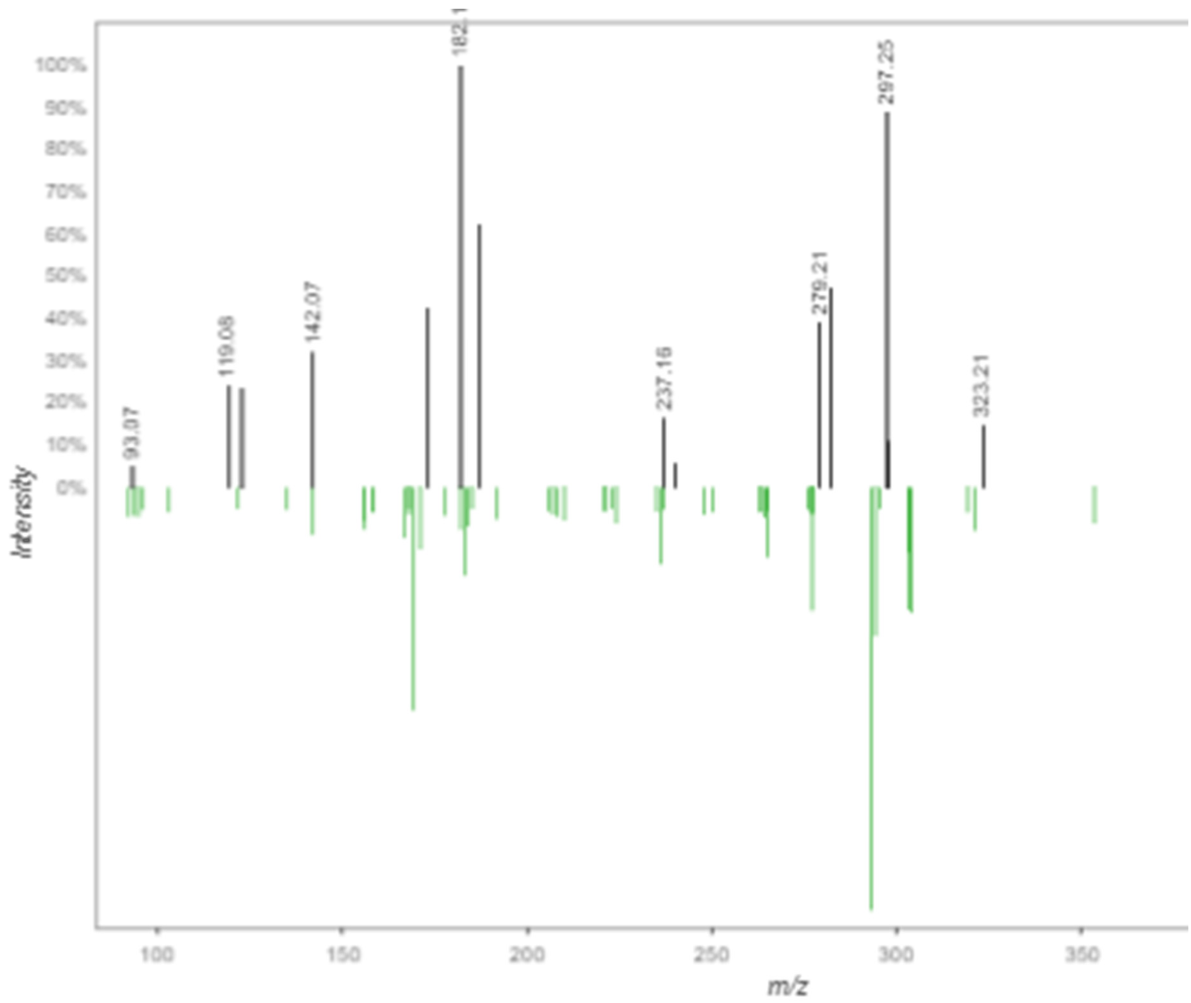

Figure S18: MS/MS match between GNPS database (green) and *Eunicella cavolini* sample (black) for compound 13.

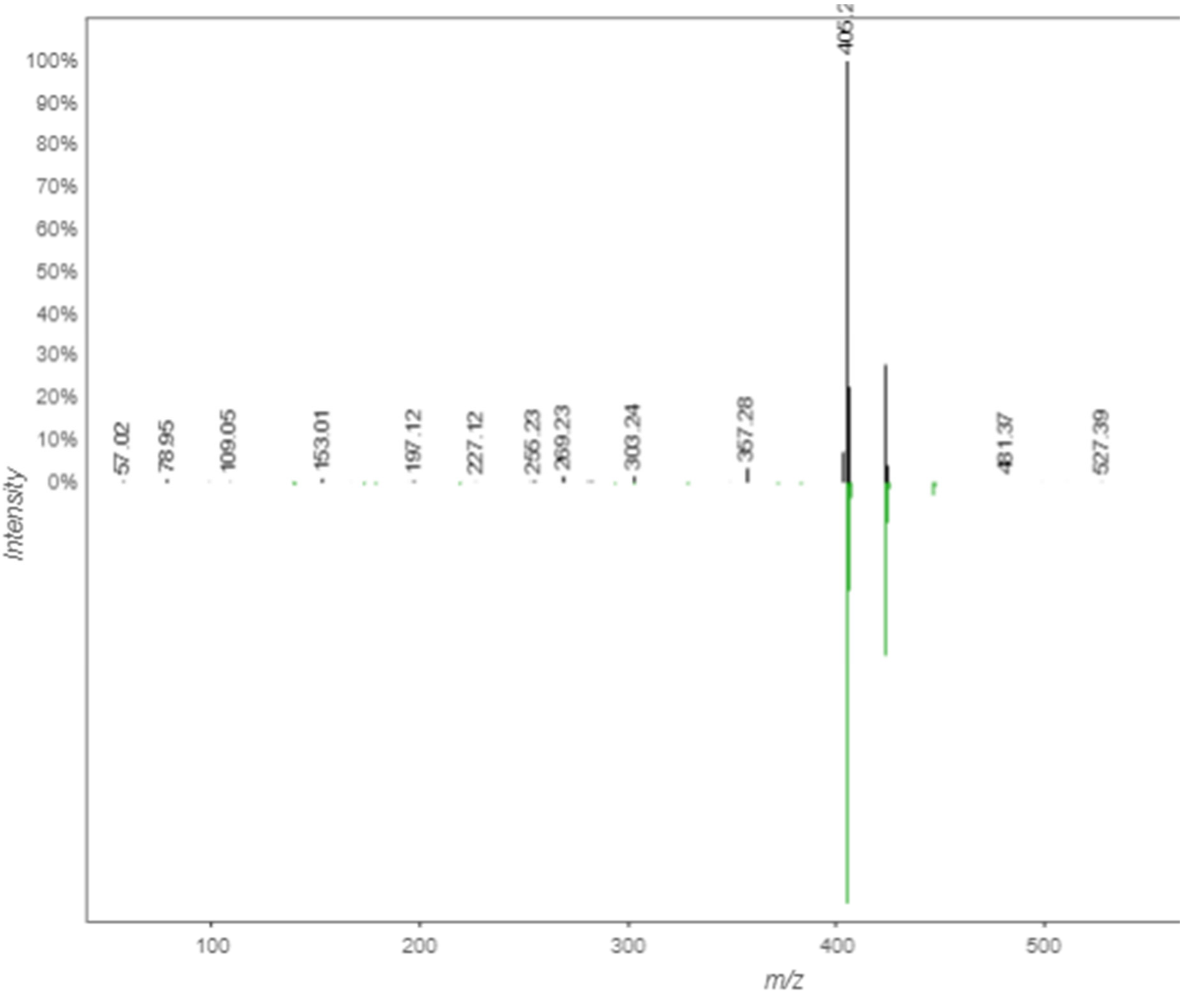

Figure S19: MS/MS match between GNPS database (green) and *Eunicella cavolini* sample (black) for compound 14.

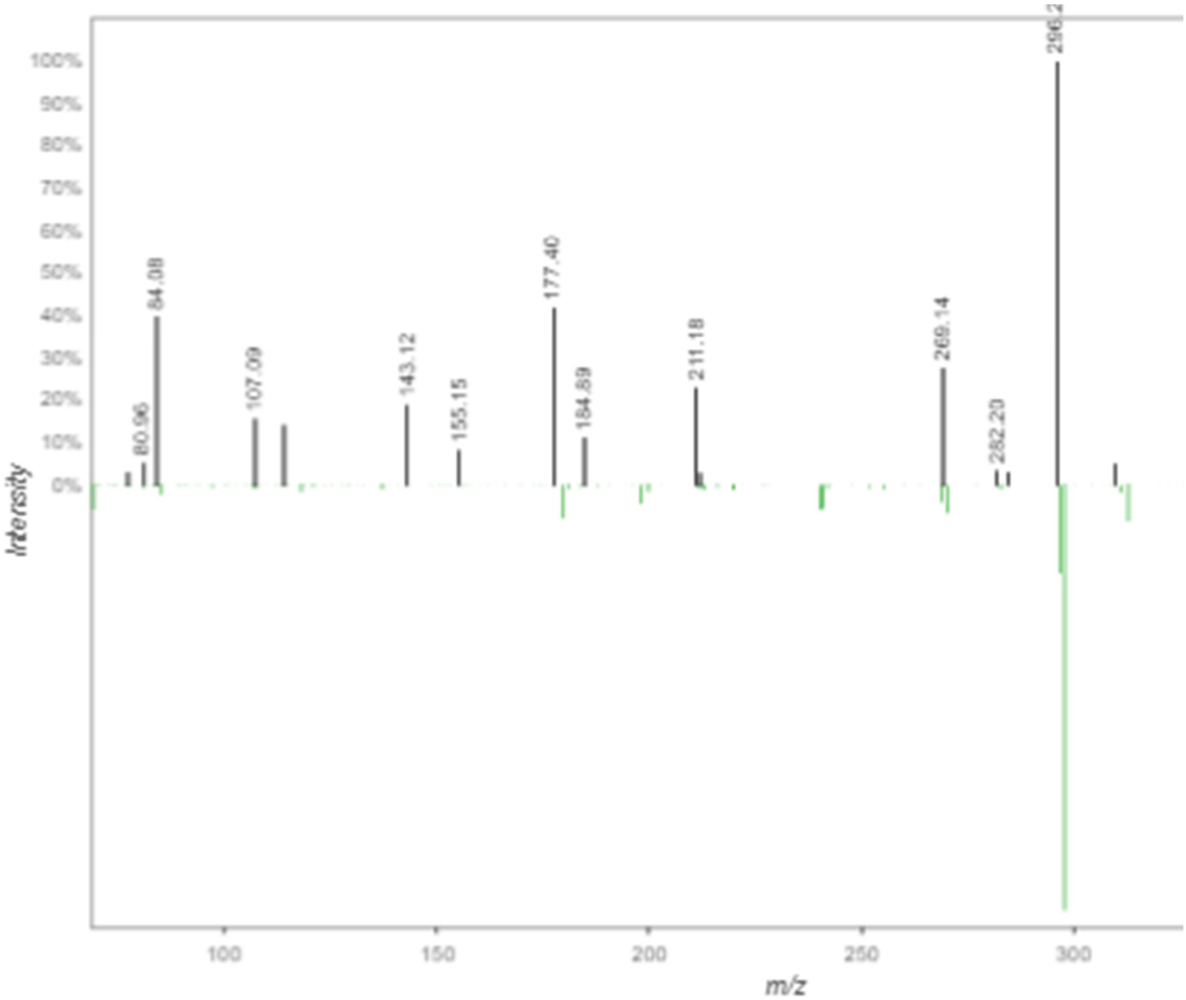

Figure S20: MS/MS match between GNPS database (green) and *Eunicella cavolini* sample (black) for compound 15.
